# Pathogenic *NR2F1* variants cause a developmental ocular phenotype recapitulated in a mutant mouse model

**DOI:** 10.1093/braincomms/fcab162

**Published:** 2021-07-20

**Authors:** Neringa Jurkute, Michele Bertacchi, Gavin Arno, Chiara Tocco, Ungsoo Samuel Kim, Adam M Kruszewski, Robert A Avery, Emma C Bedoukian, Jinu Han, Sung Jun Ahn, Nikolas Pontikos, James Acheson, Indran Davagnanam, Richard Bowman, Marios Kaliakatsos, Alice Gardham, Emma Wakeling, Ngozi Oluonye, Maddy Ashwin Reddy, Elaine Clark, Elisabeth Rosser, Patrizia Amati-Bonneau, Majida Charif, Guy Lenaers, Isabelle Meunier, Sabine Defoort, Catherine Vincent-Delorme, Anthony G Robson, Graham E Holder, Luc Jeanjean, Antonio Martinez-Monseny, Mariona Vidal-Santacana, Chloé Dominici, Cedric Gaggioli, Nadia Giordano, Matteo Caleo, Grant T Liu, Genomics England Research Consortium, Andrew R Webster, Michèle Studer, Patrick Yu-Wai-Man

**Affiliations:** 1 Moorfields Eye Hospital NHS Foundation Trust, London, UK; 2 Institute of Ophthalmology, University College London, London, UK; 3 Université Côte d’Azur, CNRS, Inserm, iBV, Nice, France; 4 Kim's Eye Hospital, Seoul, South Korea; 5 Department of Neurology, Hospital of the University of Pennsylvania, Perelman School of Medicine, Philadelphia, PA, USA; 6 Division of Ophthalmology, Children’s Hospital of Philadelphia, Philadelphia, PA, USA; 7 Department of Neurology, Perelman School of Medicine, Philadelphia, PA, USA; 8 Department of Ophthalmology, Perelman School of Medicine, Philadelphia, PA, USA; 9 Roberts Individualized Medical Genetics Center, Children’s Hospital of Philadelphia, Philadelphia, PA, USA; 10 Institute of Vision Research, Department of Ophthalmology, Gangnam Severance Hospital, Yonsei University College of Medicine, Seoul, South Korea; 11 Department of Radiology, Gangnam Severance Hospital, Yonsei University College of Medicine, Seoul, South Korea; 12 National Hospital for Neurology and Neurosurgery, University College London Hospitals NHS Trust, London, UK; 13 Department of Brain Repair & Rehabilitation, UCL Queen Square Institute of Neurology, London, UK; 14 Department of Ophthalmology, Great Ormond Street Hospital for Children NHS Foundation Trust, London, UK; 15 Paediatric Neurology, Great Ormond Street Hospital for Children NHS Foundation Trust, London, UK; 16 North West Thames Regional Genetics Service, Northwick Park Hospital, Harrow, UK; 17 North East Thames Regional Genetics Service, Great Ormond Street Hospital for Children NHS Foundation Trust, London, UK; 18 Wolfson Neurodisability Service, Great Ormond Street Hospital NHS Foundation Trust, London, UK; 19 Royal London Hospital, Barts Health NHS Trust, London, UK; 20 Department of Neuroscience, Great Ormond Street Hospital for Children NHS Foundation Trust, London, UK; 21 Department of Clinical Genetics, Great Ormond Street Hospital for Children NHS Foundation Trust, London, UK; 22 MitoLab Team, UMR CNRS 6015 - INSERM U1083, Institut MitoVasc, Angers University and Hospital, Angers, France; 23 Department of Biochemistry and Genetics, University Hospital Angers, Angers, France; 24 Genetics and Immuno-cell Therapy Team, Mohammed First University, Oujda, Morocco; 25 National Center for Rare Diseases, Inherited Sensory Disorders, Gui de Chauliac Hospital, Montpellier, France; 26 Institut des Neurosciences de Montpellier, INSERM INSERM U1051, Université de Montpellier, Montpellier, France; 27 Service d'exploration de la vision et neuro-ophtalmologie, CHRU de Lille, Lille, France; 28 Service de Génétique médicale, Hôpital Jeanne de Flandre, CHRU de Lille, Lille, France; 29 Yong Loo Lin School of Medicine, Department of Ophthalmology, National University of Singapore, Singapore, Singapore; 30 Department of Ophthalmology, University Hospital of Nimes, Nimes, France; 31 Genetic and Molecular Medicine Department, Hospital Sant Joan de Déu, Barcelona, Spain; 32 Department of Ophthalmology, Hospital Sant Joan de Déu, Barcelona, Spain; 33 University Côte d'Azur, CNRS UMR7284, INSERM U1081, Institute for Research on Cancer and Aging, Nice, France; 34 Neuroscience Institute-CNR, Pisa, Italy; 35 Cambridge Eye Unit, Addenbrooke’s Hospital, Cambridge University Hospitals, Cambridge, UK; 36 John van Geest Centre for Brain Repair and MRC Mitochondrial Biology Unit, Department of Clinical Neurosciences, University of Cambridge, Cambridge, UK

**Keywords:** *NR2F1*, inherited optic neuropathy, BBSOAS, mouse model, optic nerve head anomalies

## Abstract

Pathogenic *NR2F1* variants cause a rare autosomal dominant neurodevelopmental disorder referred to as the Bosch–Boonstra–Schaaf Optic Atrophy Syndrome. Although visual loss is a prominent feature seen in affected individuals, the molecular and cellular mechanisms contributing to visual impairment are still poorly characterized. We conducted a deep phenotyping study on a cohort of 22 individuals carrying pathogenic *NR2F1* variants to document the neurodevelopmental and ophthalmological manifestations, in particular the structural and functional changes within the retina and the optic nerve, which have not been detailed previously. The visual impairment became apparent in early childhood with small and/or tilted hypoplastic optic nerves observed in 10 cases. High-resolution optical coherence tomography imaging confirmed significant loss of retinal ganglion cells with thinning of the ganglion cell layer, consistent with electrophysiological evidence of retinal ganglion cells dysfunction. Interestingly, for those individuals with available longitudinal ophthalmological data, there was no significant deterioration in visual function during the period of follow-up. Diffusion tensor imaging tractography studies showed defective connections and disorganization of the extracortical visual pathways. To further investigate how pathogenic *NR2F1* variants impact on retinal and optic nerve development, we took advantage of an *Nr2f1* mutant mouse disease model. Abnormal retinogenesis in early stages of development was observed in *Nr2f1* mutant mice with decreased retinal ganglion cell density and disruption of retinal ganglion cell axonal guidance from the neural retina into the optic stalk, accounting for the development of optic nerve hypoplasia. The mutant mice showed significantly reduced visual acuity based on electrophysiological parameters with marked conduction delay and decreased amplitude of the recordings in the superficial layers of the visual cortex. The clinical observations in our study cohort, supported by the mouse data, suggest an early neurodevelopmental origin for the retinal and optic nerve head defects caused by *NR2F1* pathogenic variants, resulting in congenital vision loss that seems to be non-progressive. We propose *NR2F1* as a major gene that orchestrates early retinal and optic nerve head development, playing a key role in the maturation of the visual system.

## Teaser

Jurkute et al. expands the phenotypic spectrum of pathogenic *NR2F1* variants, providing a detailed characterization of the retinal and optic nerve changes with high-resolution optical coherence tomography imaging. Data from a mutant mouse model confirms the key role played by NR2F1 in directing retinogenesis and the development of the optic nerve head.

## Graphical Abstract

**Graphical Abstract:**
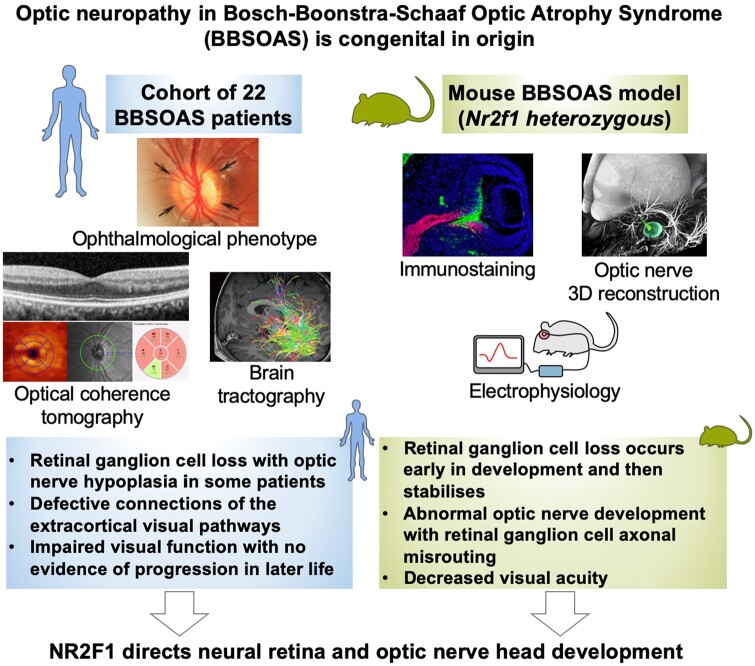

## Introduction

Inherited optic neuropathies are an important cause of visual impairment in young children with an estimated prevalence of 1 in 10 000.^1^ Although genetically heterogeneous with both nuclear and mitochondrial genes being implicated, the pathological hallmark is the marked vulnerability of retinal ganglion cells (RGCs) leading to optic nerve degeneration and irreversible visual loss.^2^ In autosomal dominant or recessive optic atrophy caused by pathogenic *OPA1* (OMIM 605290) and *WFS1* (OMIM 606201) variants, progressive RGC loss starts in early childhood and most patients are registered legally blind by the fifth decade of life.^3^ High-resolution optical coherence tomography (OCT) imaging has made it possible to visualize and monitor the loss of RGCs, and in most inherited optic neuropathies, there is early loss of RGCs within the papillomacular bundle that becomes more generalized as the disease progresses.^1^^,^^4^ Optic nerve hypoplasia (ONH) is a non-progressive congenital disease characterized by underdevelopment of the optic nerve that is often accompanied by other structural ocular abnormalities. In addition, ONH is frequently associated with other neurodevelopmental abnormalities, such as brain malformations, developmental delay, intellectual disability and autism spectrum disorders.^5–7^ Genetically, variants in genes involved in transcriptional regulation, chromatin remodelling, scaffolding proteins and MAPK signalling pathway have been associated with ONH. The transcription factors implicated in ONH participate in the proper development of the optic stalk (OS) and optic nerve by directly participating in the intricate sequential steps coordinating ocular morphogenesis and maturation.^8^

Bosch–Boonstra–Schaaf Optic Atrophy Syndrome (BBSOAS) (OMIM 615722; ORPHA 401777) is an autosomal dominant disorder characterized by delayed neurodevelopment, moderate to severe intellectual disability and visual impairment.^9^ BBSOAS is caused by pathogenic variants in the *NR2F1* gene (OMIM 132890, 5q15), which encodes a conserved orphan nuclear receptor protein acting as a strong transcriptional regulator. NR2F1 represents an evolutionarily highly conserved protein,^10^ with a classic nuclear receptor structure and two highly conserved domains: the functional DNA-binding domain (DBD) and a ligand-binding domain (LBD). Structural variants spanning *NR2F1* were first reported as causing human disease in patients with neurodevelopmental syndromes characterized by mental retardation, epilepsy and deafness.^11^^,^^12^ A subsequent functional study confirmed the pathogenic nature of *NR2F1* variants and their association with syndromic optic atrophy.^9^ Further cases have since been reported that have expanded the phenotypes associated with pathogenic variants in *NR2F1* and highlighted the marked variability in disease severity.^13–18^ Visual impairment is one of the major features described in BBSOAS with patients developing ocular, visual pathway and cortical visual impairment in some cases. However, the structural and functional defects within the retina and the optic nerve need to be defined further to provide greater insight into the pathophysiological mechanisms of the disease, in particular, whether the defects are congenital in origin and/or whether it is a progressive neurodegenerative process occurring after birth.

A knockout *Nr2f1* mouse model has recently been characterized highlighting the importance of Nr2f1 in regulating the early development of the visual system, including the formation of the optic cup and optic nerve.^19^ We therefore capitalized on the availability of such a mouse model to further delineate the post-natal ocular expression pattern of Nr2f1 and to investigate the deleterious consequences of Nr2f1 loss on retinal and optic nerve development and maturation. To complement these experiments, we also performed a detailed characterization of NR2F1 in human foetal retina. In parallel, we conducted a deep phenotyping study of 22 patients, including familial cases, carrying a pathogenic *NR2F1* variant with structural OCT imaging and electrophysiological evaluation of visual function. The findings suggest a neurodevelopmental basis for the observed visual impairment associated with *NR2F1* variants, rather than the progressive RGC loss described in classical inherited optic neuropathies. The use of a mutant *Nr2f1* mouse model to explore this hypothesis provided concordant observations, confirming that the loss of RGCs and axonal misguidance in early development leads to ONH and the development of optic atrophy, resulting in impaired visual acuity, and with the severity of the structural defects depending on the residual amount of wild-type protein.

## Materials and methods

### Study cohort

In this retrospective multicentre study, the clinical information of individuals with confirmed pathogenic *NR2F1* variants was reviewed to establish the phenotypic spectrum. The initial cohort of three *NR2F1*-positive families was identified at Moorfields Eye Hospital NHS Foundation Trust (London, UK), with the contribution of additional families from Great Ormond Street Hospital for Children NHS Foundation Trust (London, UK), the Children's Hospital of Philadelphia (Philadelphia, USA), Gangnam Severance Hospital (Seoul, South Korea), Hospital Sant Joan de Déu (Barcelona, Spain), University Hospitals of Nîmes, Lille and Montpellier (France). The recruitment centres interrogated their institutional clinical databases for individuals with genetically confirmed *NR2F1* variants. Two of these individuals, NR2F1_10 (South Korea) and NR2F1_4 (France), have previously been reported.^18^^,^^20^ This retrospective study adhered to the tenets of the Declaration of Helsinki and the contributing study centres had the relevant ethical and institutional approvals.

### Clinical phenotyping

**Figure 1:**
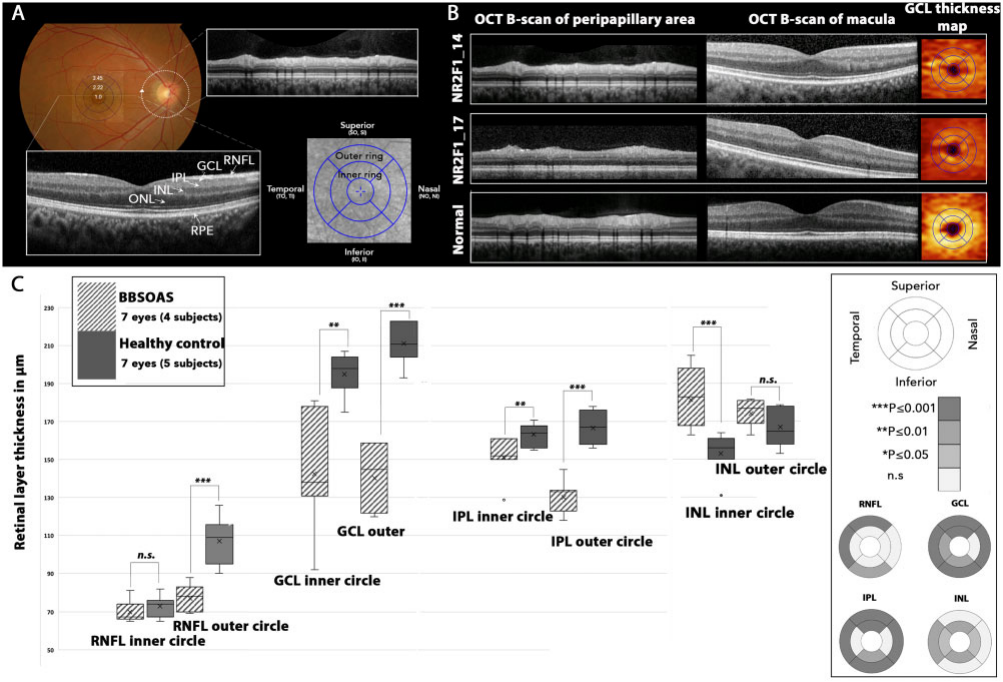
**Optical coherence tomography (OCT) composite**. (**A**) Schematic representation of method used to analyse SD-OCT scans (normal eye). Macula grid of central (1.0 mm diameter), inner (2.22 mm diameter) and outer rings (3.45 mm diameter) was centred on the fovea. The 9 sectors in the grid represent the areas where the thickness of the retinal layers (RNFL, GCL, IPL, INL, ONL and RPE) were measured. Circular scan was used to evaluate parapapillary RNFL thickness. The arrow indicates the start position. (**B**) OCT profiles of two affected individuals and normal control for comparison (single right eye selected). OCT B-scan of the peripapillary area showing preserved RNFL layer. OCT B-scan through the macula showing thinning of the GCL. GCL thickness map indicates thinning of GCL in two affected individuals. (**C**) Comparison of the thickness of four retinal layers within the inner and outer circles, and in nine sectors for individuals with *NR2F1* variants (pattern fill) and normal controls (grey) subjects. The grey scale indicates significant differences in retinal layer thickness between the *NR2F1* and control groups. The Mann–Whitney U-test was used to for statistical analysis. *P ≤ 0.05; **P ≤ 0.01; ***P ≤ 0.001; n.s., non-significant. GCL, ganglion cell layer; II, inferior inner; INL, inner nuclear layer; IO, inferior outer; IPL, inner plexiform layer; NI, nasal inner; NO, nasal outer; ONL, outer nuclear layer; RNFL, retinal nerve fibre layer; RPE, retinal pigment epithelium; SI, superior inner; SO, superior outer; TI, temporal inner; TO, temporal outer.

All affected individuals underwent a comprehensive neuro-ophthalmological examination during the initial diagnostic workup or following the identification of a pathogenic *NR2F1* variant. When feasible, fundus imaging and high-resolution spectral-domain Spectralis OCT imaging (Heidelberg Engineering Inc., Heidelberg, Germany) was performed. Segmentation was obtained using the automated segmentation software for the Spectralis OCT device (Heidelberg Engineering, software version 1.9.10.0). Each macular OCT scan was inspected to assess the accuracy of the automated segmentation in the determination of retinal thickness. Manual adjustments were made when needed. Retinal thickness maps were overlapped with macula grid (1.0, 2.22, 3.45 mm diameters) on OCT scans (Fig. 1A–C). The central 1 mm diameter circle was defined as the central subfield, followed by the inner 2.22 mm diameter ring and the outer 3.45 mm diameter ring. Both rings were divided into the inner and outer superior, nasal, inferior and temporal sectors, and average values were calculated per sector. The built-in software was used to automatically calculate separate thickness values for the retinal nerve fibre layer (RNFL), retinal ganglion cell layer (GCL), inner plexiform layer (IPL), inner nuclear layer (INL), outer nuclear layer (ONL), and retinal pigment epithelium (RPE) in the inner and outer (global) circles, and for each sector. Eyes with bad quality scans were excluded. The data obtained were compared with age-matched healthy controls. Macular normative data were generated from SD-OCT images of 7 normal eyes from 5 individuals with no known retinal or optic nerve disease, and best-corrected visual acuity (BCVA) of 0.00 logMAR.

Electrophysiological investigation was available for 12 individuals, including pattern visual evoked potential (PVEP, *N* = 9), flash VEP (FVEP, *N* = 11), pattern electroretinography (PERG, *N* = 6) and full-field electroretinography (ERG, *N* = 7), which were performed to incorporate the International Society for Clinical Electrophysiology of Vision (ISCEV) standards.^21–23^

White matter tractography was performed on one subject (NR2F1_10) to assess the functional connectivity of the visual pathway. Using the occipital pole and primary visual cortex as a seed point, the inferior frontal–occipital fasciculus, the superior longitudinal fasciculus, and the inferior longitudinal fasciculus were reconstructed. High angular resolution diffusion images were acquired using a spin-echo EPI sequence with 3 multi-slice acceleration (TE 65 ms, TR 3200 ms, 64 directions, Bmax 3000, Bmin 0, 2 mm isotropic voxel size). White matter fibre tracking and reconstruction were performed using DSI Studio software (http://dsi-studio.labsolver.org/ last accessed July 2021) with a sparse solution of fibre orientation distribution function by diffuse decomposition. Termination criteria were based on a threshold of quantitative anisotropy of 1.7 and a conservative angle change of >45˚.

### Molecular genetic analysis

**Figure 2:**
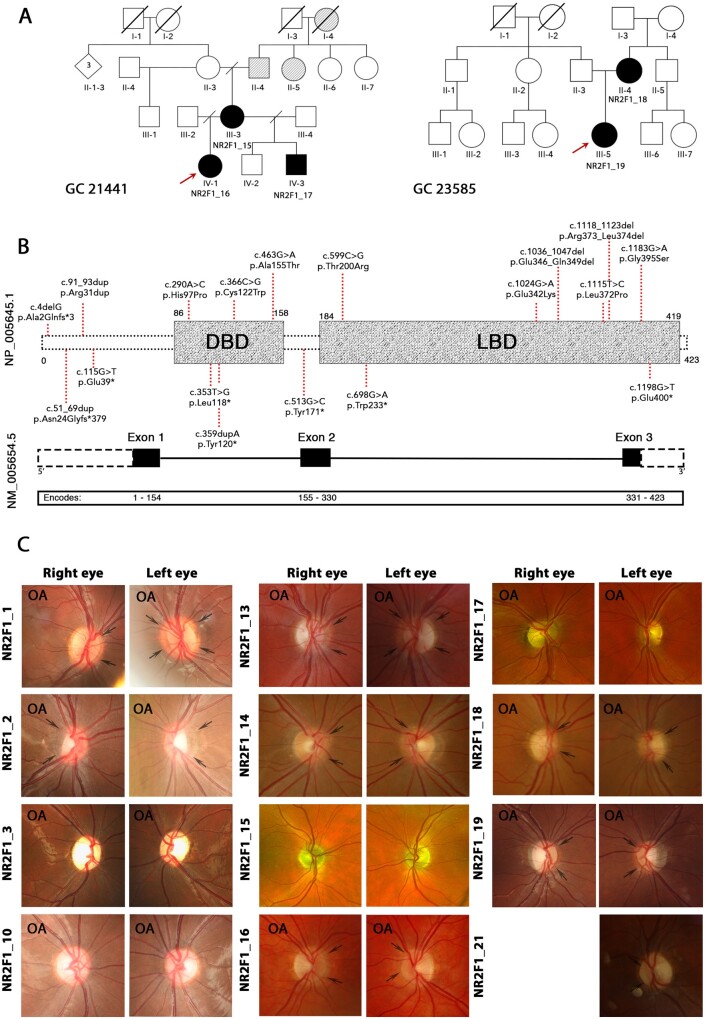
**Genotype spectrum and optic nerve head imaging of study subjects**. (**A**) Pedigrees of familial cases identified in our *NR2F1* patient cohort. Red arrows indicate the probands of the two familial cases (GC 21441 and GC 23585). Black shading indicates affected individuals. Grey shading denotes a history of poor vision in family members who have not been assessed formally. (**B**) Schematic representation of the *NR2F1* gene with three exons (black boxes) and the NR2F1 protein structure showing two domains (grey boxes). (**C**) Colour photographs of the optic nerve heads demonstrating optic atrophy and/or optic nerve hypoplasia. Black arrows highlight the double-ring sign. OA—indicates optic nerve heads with clinical signs of optic atrophy. The locations of the *NR2F1* variants identified in our study cohort are indicated in red. DBD, DNA binding domain; LBD, ligand binding domain.

As part of the Genomic England 100 000 Genomes Project, both pilot and main studies,^24^ individuals NR2F1_14 (GC22770, UK), NR2F1_16 (GC21441, UK), NR2F1_18 (GC23585, UK) and NR2F1_19 (GC23585, UK) from three independent families diagnosed with inherited optic neuropathy were analysed by whole-genome sequencing. A multistep rare variant filtering pipeline was employed, as previously described, to identify the most likely disease-causing variant.^25^ As autosomal dominant optic atrophy was suspected (Fig. 2A), a minor allele frequency threshold of <0.001 was used for all coding variants occurring in a virtual panel of genes previously shown to be associated with inherited optic neuropathies (https://panelapp.genomicsengland.co.uk/panels/186/ last accessed July 2021). A similar analysis was performed on whole-genome sequencing data from individuals in the Genomic England 100 000 Genomes Project to identify additional individuals carrying pathogenic *NR2F1* variants. The search was performed in October 2019.

Case NR2F1_2 (South Korea) was identified by whole-exome sequencing using Twist Human Core Exome kit (Twist Bioscience, San Francisco, CA, USA). A 429 targeted genes panel sequencing was performed in NR2F1_3 (South Korea) and NR2F1_10 (South Korea), with the latter case having been previously reported.^18^ Cases NR2F1_1 (Spain), NR2F1_13 (France) and NR2F1_21 (France) were identified by next-generation sequencing (NGS). For NR2F1_1 (Spain), an NGS panel of 6710 genes associated with pathologies described in the Human Gene Mutation Database, GeneTest.org and the OMIM catalogue was used for screening. In some cases, NextSeq500 Illumina sequencing was performed *a posteriori* for validation of *NR2F1* variants identified by the NGS panel. Cases NR2F1_13 (France) and NR2F1_21 (France) were identified using an NGS panel of 88 nuclear genes known to cause inherited optic neuropathies or disorders linked to disturbed mitochondrial dynamics. Library preparation for each sample was carried out using SureSelect Target Enrichment System for Sequencing on Ion Proton (Manuel number G7530-90005). Sample emulsion PCR, emulsion breaking and enrichment were performed using the Ion PI™ Chip Kit v2 BC (Cat. no. 4484270) and Ion PI™ IC 200 Kit (Cat. no. 4488377) and sequencing was undertaken using sequencing with the Ion Proton™ System. The cases from the Children's Hospital of Philadelphia (USA) were all identified by performing whole-exome sequencing, except NR2F1_22 (USA) who underwent single-gene testing.

All the *NR2F1* variants in the study cohort, and their segregation if available, were confirmed by Sanger sequencing. The Genome Aggregation Database (gnomAD, https://gnomad.broadinstitute.org last accessed July 2021) was used to assess for the rarity of the variants in the general population. *In silico* analysis was performed to evaluate the likely impact on protein function using Polymorphism Phenotyping v2 (http://genetics.bwh.harvard.edu/pph2/ last accessed July 2021) and Mutation Taster (http://www.mutationtaster.org/ last accessed July 2021) predictive algorithms. The evolutionary conservation of the affected amino acid residues across orthologues was assessed using Uniprot sequence alignments (https://www.uniprot.org/help/sequence-alignments last accessed July 2021).

### Animal procedures

All mouse experiments were conducted in accordance with the relevant national and international guidelines and regulations (European Union rules; 2010/63/UE), and with approval by the local ethical committee in France (CIEPAL NCE/2019–548). *Nr2f1* heterozygous (*HET*) and homozygous (*KO*) mice were generated and genotyped as previously described.^26^ Littermates of *HET* and *KO* mice with normal *Nr2f1* alleles were used as control wild-type mice (*WT*). Midday of the day of the vaginal plug was considered as embryonic day 0.5 (E0.5). Control and mutant mice were bred in a 129S2/SvPas background. Both male and female embryos and adults were used in this study, with the age being specified for each specimen used in specific experiments. Standard housing conditions were approved by the local ethical committee in France (CIEPAL NCE/2019–548). Briefly, adult mice were kept on a 12-h light–dark cycle and three animals were housed per cage with the recommended environmental enrichment (wooden cubes, cotton pad and igloo), and with food and water *ad libidum*. The protocols for immunofluorescence (IF), *in situ* hybridization, Western blot, intracortical murine visual evoked potential (VEP) recording and three-dimensional (3D) imaging of mouse tissues have been provided in the Supplementary Appendix.

### Collection and processing of human samples

Cryostat section of non-pathological human foetuses was kindly provided by Cécile Allet and Paolo Giacobini (Lille, France; Agence de la Biomédecine, Saint-Denis la Plaine, France, protocol n°: PFS16-002), while GW24 and GW34 paraffin eye sections were provided by Fabien Guimiot (INSERM U1141, Hôpital Robert Debré, Paris, France). All experiments involving the use of human samples conformed to the principles set out in the WMA Declaration of Helsinki and the Department of Health and Human Services Belmont Report. Tissues were made available in accordance with French bylaws (Good practice concerning the conservation, transformation and transportation of human tissue to be used therapeutically, published on 29 December 1998). For GW14 sections, a non-pathological human foetus (14 gestational weeks, *n* = 1) was obtained from a voluntarily terminated pregnancy after obtaining written informed consent from the parents (Gynaecology Department, Jeanne de Flandre Hospital, Lille, France). The foetus was fixed by immersion in 4% PFA at 4°C for 7 days. The tissues were then cryoprotected in 30% sucrose/PBS for 3 days, embedded in Tissue-Tek OCT compound (Sakura Finetek, USA), frozen in dry ice and stored at −80°C until sectioning. Frozen samples were cut serially at 20 µm using a Leica CM 3050S cryostat (Leica Biosystems Nussloch GmbH, Germany).

### Statistical analysis

All data were statistically analysed and graphically represented using Microsoft Office Excel software (Version 2003), IBM SPSS Statistics Software (Version 26) and GraphPad Prism (Version 7.00). Quantitative data are shown as the mean ± SEM. For cell percentage/number quantification after IF, measurements were performed on at least 5 sections coming from 2 to 3 different animals, unless otherwise stated. Fixed embryos/eyes with damaged tissues were excluded from any further analysis/processing. Microscope images were processed with Photoshop or ImageJ software, by randomly overlapping fixed-width (100 µm) rectangular boxes on the area of interest [e.g. sectioned neural retina (NR)], then quantifying positive cells inside the boxes. When calculating percentages over the total cell number, the latter was quantified by counting DAPI^+^ nuclei, unless otherwise specified. Data were analysed using the Mann–Whitney U-test or two-tailed Student’s *t*-test (when comparing two data groups), or by two-way analysis of variance (ANOVA) for comparison of three or more groups. Statistical significance was set as follows: *P ≤* *0.05; **P ≤* *0.01; ***P ≤* *0.001.

### Data availability

The anonymized data that support the findings of this study can be requested from the corresponding author.

## Results

### Pathogenic NR2F1 variants are clustered within the DBD and LBD

A total of 19 *NR2F1* variants were identified in the study’s patient cohort, all of which are absent from the gnomAD dataset (Table 1). Three variants, namely, c.290 A > C p.(His97Pro), rs1554074673; c.353 T > G p.(Leu118*), rs1561523796; and c.1115T > C p.(Leu372Pro), rs1554075105, were found in ClinVar database. The majority of *NR2F1* variants (13/19, 59.1%) are located within the DBD and LBD (Fig. 2B). Nine (40.9%) individuals carried frameshift, stop-gain variants or entire gene deletion, whereas 13 (59.1%) individuals carried missense variants or small deletions/duplications. The identified *NR2F1* variants were predicted to be disease causing based on *in silico* analysis. Multiple alignment of human NR2F1 orthologues confirmed the strictly conserved nature of the protein across different species (Supplementary Fig. 1). In addition, we report, for the first time, two familial *NR2F1* cases. The identified variants [c.1115T>C p.(Leu372Pro) and c.1118_1123del p.(Arg373_Leu374del)] co-segregated with disease status in other affected family members (Fig. 2A). In the majority of families (14/19, 73.7%), the *NR2F1* variants were found to be *de novo* or likely *de novo*.

**Table 1:** *NR2F1* variants identified in the patient cohort

Subject	Sex	HGVs	HGVp	Genotype	Domain	Type	In silico:
							Polyphen-2, Mutation taster
							(CADD score)
NR2F1_1	F	c.4delG	p.(Ala2Glnfs*3)	Het		de novo	Disease causing (27.5)
NR2F1_2	F	c.51_69dup	p.(Asn24Glyfs*379)	Het		unknown	NA (24)
NR2F1_3	M	c.91_93dupCGC	p.(Arg31dup)	Het		unknown	NA (18.67)
NR2F1_4	F	c.115G>T	p.(Glu39*)	Het		de novo	Disease causing (34)
NR2F1_5	M	c.290A>C ^a^^, b^	p.(His97Pro)	Het	DBD	de novo	Probably damaging, disease causing (26.5)
NR2F1_6	M	c.353T>G ^b^	p.(Leu118*)	Het	DBD	de novo	Disease causing (36)
NR2F1_7	M	c.359dupA	p.(Tyr120*)	Het	DBD	likely de novo	Disease causing (33)
NR2F1_8	F	c.366C>G ^a^	p.(Cys122Trp)	Het	DBD	de novo	Probably damaging, disease causing (28.7)
NR2F1_9	M	c.463G>A ^a^	p.(Ala155Thr)	Het	DBD	de novo	Disease causing (34)
NR2F1_10	M	c.513G>C ^a^	p.(Tyr171*)	Het		unknown	Disease causing (37)
NR2F1_11	M	c.599C>G	p.(Thr200Arg)	Het	LBD	de novo	Probably damaging, disease causing (23.2)
NR2F1_12	M	c.698G>A	p.(Trp233*)	Het	LBD	de novo	Disease causing (37)
NR2F1_13	F	c.1024G>A	p.(Glu342Lys)	Het	LBD	de novo	Probably damaging, disease causing (32)
NR2F1_14	F	c.1036_1047del	p.(Glu346_Gln349del)	Het	LBD	likely de novo	Disease causing (22.6)
NR2F1_15	F						
NR2F1_16	F	c.1115T>C ^a^^, b^	p.(Leu372Pro)	Het	LBD	familial	Probably damaging, disease causing (32)
NR2F1_17	M						
NR2F1_18	F	c.1118_1123del	p.(Arg373_Leu374del)	Het	LBD	familial	Disease causing (22.7)
NR2F1_19	F						
NR2F1_20	M	c.1183G>A	p.(Gly395Ser)	Het	LBD	de novo	Probably damaging, disease causing (32)
NR2F1_21	M	c.1198G>T	p.(Glu400*)	Het	LBD	de novo	Disease causing (41)
NR2F1_22^c^	F	∼599kb deletion	5q15 deletion (92,914,091–93,513,068)	Het	WGD	de novo	NA

CADD, Combined Annotation Dependent Depletion; DBD, DNA binding domain; Het, heterozygous; LBD, ligand binding domain; NA, not available; WGD, whole gene deletion.

a Previously reported variants.

b Variants listed in Clinvar database.

c ∼599 kb deletion includes entire NR2F1, FAM172A genes and partial NR2F1-AS1 and last exon of KIAA0825.

### Visual function in individuals carrying pathogenic NR2F1 variants

Clinical data were available for 22 patients from 19 independent families, with 20 patients not having been reported previously. The mean age at the last ophthalmological assessment was 15.2 years (SEM = 2.7 years, range = 1.5–49.0 years). There was an equal sex distribution with 11 women and 11 men. Vision impairment and optic nerve head pathology were identified when individuals were examined following referrals for nystagmus, strabismus, poor fixation and/or concerns about neurodevelopmental progress. Impairment of vision was noticed in early childhood in all individuals, except for case NR2F1_15 (UK), who was found to have subnormal vision in her 50 s (Table 2). However, that particular individual had other classical features described in BBSOAS, namely, delay in walking and speaking, and mildly dysmorphic facial features. In addition to optic atrophy, all affected individuals developed other neuro-ophthalmological and systemic deficits (Supplementary Table 1).

**Table 2:** Summary of ophthalmological features in individuals carrying heterozygous *NR2F1* variants

Subject	OA (AAD)	ONH	CVI	**BCVA** **RE/LEa**	Squint	Nystagmus	Refractive Error	Visual Electrophysiology	OCT
								(Bilateral)	(Bilateral)
NR2F1_1	Yes (8 years)	Yes	ND	0.4/0.5	Yes	No	H (Mild)	ND	ON: RNFL thinning; Macula: ND
NR2F1_2	Yes	Yes	Yes	1.3/1.3	No	Yes	M (Mild); A	ND	ON: mild RNFL thinning; Macula: GCL thinning
NR2F1_3	Yes (1 year)	No	Yes	0.1/0.7	Yes	Yes	H (Mild)	ON dysfunction	ON: RNFL thinning; Macula: RNFL, GCL thinning
NR2F1_4	Yes	No	ND	0.5/0.4	Yes	No	H (Moderate/high); A	ND	ON: RNFL thinning; Macula: RNFL, GCL thinning
NR2F1_5	Yes (3 months)	ND	No	1.43/1.7	No	No	H (Mild)	ND	ND
NR2F1_6	Yes (33 months)	No	No	0.7/0.7	Yes	Yes	H (Moderate)	ND	ND
NR2F1_7	Yes (14 years)	ND	No	0/0.1	Yes	No	E	ND	ND
NR2F1_8	Yes (9 months)	ND	Yes	0.98/0.98	Yes	No	H (Moderate); A	ND	ND
NR2F1_9	No	No	Yes	0.62/0.60	Yes	No	H (Moderate); A	ON dysfunction	ND
NR2F1_10	Yes	No	Yes	0.7/0.7	No	Yes	E	Inconclusive due to poor cooperation	ON: RNFL thinning; Macula: RNFL, GCL thinning
NR2F1_11	No	ND	No	NR	Yes	No	H (High); A	ND	ND
NR2F1_12	Yes	No^b^	Yes	ND	Yes	No	H (Mild); A	ON dysfunction	ND
NR2F1_13	Yes (7 years)	Yes	No	0.6/0.9	Yes	Yes	H (Moderate/high); A	ON dysfunction	ON: RNFL thinning; Macula: RNFL, GCL thinning
NR2F1_14	No	Yes	Yes	0.43/0.43	No	Yes	M (Mild)	No definite evidence of ON/RGC dysfunction	ON: normal; Macula: Mild GCL thinning
NR2F1_15	No	No^b^	Yes	0.3/0.18	Yes	No	H (grade unknown)	ON/RGC dysfunction	ON: normal; Macula: Mild GCL thinning
NR2F1_16	Yes	Yes	Yes	1.48/1.48	Yes	Yes	M (Mild)	ON/post-retinal dysfunction	ON: RNFL thinning; Macula: RNFL, GCL thinning
NR2F1_17	Yes	No	Yes	0.78/1.0^c^	Yes	Yes	H (High)	ON/RGC dysfunction	ON: RNFL thinning; Macula: RNFL, GCL thinning
NR2F1_18	Yes	Yes	No	0.22/0.22	Yes	No	M (Mild)	ON/RGC dysfunction	ON: RNFL thinning; Macula: ND
NR2F1_19	Yes	Yes	No	0.3/0.48	Yes	Yes	M (Moderate/high)	ON dysfunction with possible additional right retro-chiasmal dysfunction	ON: RNFL thinning; Macula: RNFL, GCL thinning
NR2F1_20	No	No	Yes	0.4/0.4	Yes	No	H (grade unknown)	Evidence of macular and visual pathway dysfunction	ND
NR2F1_21	Yes (2 years)	Yes	No	1.0/1.0	No	No	H (Moderate); A	ND	Macula: Mild RNFL, GCL thinning
NR2F1_22	Yes (6 years)	ND	No	1.0/0.88	Yes	Yes	H (Mild); A	ND	ON: RNFL thinning; Macula: ND

AAD, age at diagnosis; A, astigmatism; BCVA, best-corrected visual acuity; CVI, cerebral visual impairment; E, emmetropia; GCL, ganglion cell layer; H, hyperopia; LE, left eye; M, myopia; ND, no data; NR, not recordable. Fixation was central, steady and maintained; OA, optic atrophy; OCT, optical coherence tomography; ON, ON; ONH, ON hypoplasia; RE, right eye; RGC, retinal ganglion cells; RNFL, retinal nerve fibre layer.

a BCVA at the last clinic visit (LogMAR).

b Small ON head.

c Left eye is densely amblyopic with previous unsuccessful treatment.

BCVA was available for 40 eyes from 20 individuals. The mean LogMAR visual acuity at the last ophthalmological assessment was 0.64 (SEM = 0.06, range = 0–1.70). Follow-up visual acuity data were available for 12 (63.2%) individuals (mean = 8.9 years, range = 16 months—17.0 years) (Supplementary Table 2). Four of these individuals were within the age group (up to 5 years old) when visual maturation had not yet been reached. Seven individuals (four with hyperopia, two with myopia and one with emmetropia) retained stable vision since their initial visit with each worsening being due to progression of an underlying refractive error, which was corrected with the appropriate prescription. Only one individual, NR2F1_16 (UK), experienced visual worsening over an 11-year follow-up period with LogMAR visual acuity decreasing from 0.43/0.30 at baseline to 1.48/1.48 at the last clinic visit (right eye/left eye). The latest refraction record indicated a change in refraction (patient became myopic). The majority of the study cohort (20/22, 91.0%) had a refractive error with variable degrees of hyperopia observed in 15/22 (68.2%) individuals (Table 2). The mean LogMAR BCVA was not significantly different between individuals carrying frameshift, stop-gain variants or entire gene deletion (0.70, SEM = 0.09, range = 0–1.30) compared to those with missense variants or small deletions/duplications (0.61, SEM = 0.08, range = 0.10–1.70, *P *=* *0.462). There was also no significant difference in mean LogMAR BCVA between patients with variants located within the DBD (0.78, SEM = 0.17, range = 0–1.70) and those with variants located within the LBD (0.52, SEM = 0.07, range = 0.18–1.00, *P *=* *0.103).

Twelve individuals (54.5%) underwent visual electrophysiological assessment. The original traces were available for five subjects tested at Moorfields Eye Hospital. The diagnostic reports for the remaining subjects were reviewed. The analysis of pattern reversal VEPs (PVEP) was frequently complicated by the presence of nystagmus. In two patients, there were better binocular than monocular responses in keeping with the increased nystagmus under monocular viewing conditions. Monocular PVEPs were undetectable in four subjects. In three others with available waveforms, responses were subnormal (<5 µV) and characterized either by a single positive peak of abnormally short peak time (*N* = 1; peak time 80–82 ms); a bifid waveform with abnormally early and late peaks (*N* = 1); or a polyphasic waveform also lacking a clear ‘P100’ component (*N* = 1). Abnormal FVEPs were documented in 8/10 cases. For four of the five subjects in whom the original recordings were available for review, the FVEPs had a grossly abnormal waveform with a prominent early positive peak (76–89 ms) followed by a second broad positive component (170–220 ms). No significant interocular asymmetry was present.

Pattern ERGs, being recorded binocularly, were less susceptible to nystagmus than the PVEPs. P50 components were of abnormally short peak time in three eyes of two subjects (41–44 ms; lower limit of normal = 46 ms) including both eyes of a 21-year-old individual, which showed additional reduction in the N95:P50 ratio (1.0; lower limit of normal 1.1). Four eyes of three other subjects showed asymmetry in the N95:P50 ratio, including one subject who was tested using a large checkerboard field (right eye ratio =0.9; lower limit of normal for a large stimulus field = 1.0). Three eyes showed mildly subnormal P50 components (minimum amplitude 1.7 µV; lower limit of normal = 2.0 µV), associated with a shortened P50 peak time in one. Full-field ERGs were normal in all seven cases. Three subjects had follow-up recording (intervals 1, 5 and 10 years) and none showed definite clinically significant deterioration.

### The effects of pathogenic NR2F1 variants on RGCs structure and their projections

Fundus examination revealed variable optic atrophy in 17/22 (77.3%) individuals. Ten (45.5%) individuals had small and/or tilted hypoplastic optic nerves. Colour optic nerve head photographs were available for 12 (54.6%) individuals (Fig. 2B). In 8 individuals, a yellowish peripapillary halo, the so-called double-ring sign (Fig. 2B, black arrows), was observed consistent with ONH. Proper alignment and good quality OCT scans are difficult to obtain in patients with nystagmus or behavioural disorders. OCT imaging was available for 14 (63.6%) individuals. High-resolution spectral-domain OCT macular scans were available for further analysis in 7 eyes of 4 individuals. Based on the peripapillary and macular OCT B-scans visual inspection, the RNFL thickness was relatively preserved in individuals carrying *NR2F1* variants compared with a more prominent thinning of the GCL (Fig. 1B). Macular OCT scans were used for further analysis. The mean RNFL thickness was significantly thinner in three segments, namely, the superior outer (*P *=* *0.001), inferior outer (*P *=* *0.004) and nasal outer (*P *=* *0.001) segments (Fig. 1C and Supplementary Table 3). The GCL was significantly thinner compared with normal controls in all except the temporal inner segment. A similar pattern was observed for IPL layer thickness. In contrast, the INL was significantly thicker in individuals carrying *NR2F1* variants compared with age-matched controls (Fig. 1C). One individual, NR2F1_14 (UK), had serial macular volume scans over a follow-up period of 5 years (Supplementary Fig. 2). No marked change in RNFL and GCL thickness was observed during that period.

**Figure 3:**
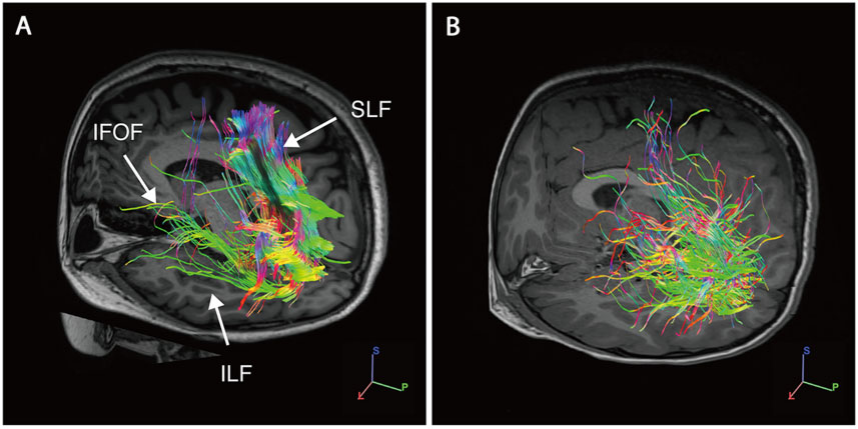
**White matter tractography with high angular resolution diffusion imaging (HARDI).** (**A**) Healthy control subject with normal vision. (**B**) Individual NR2F1_10 harbouring the c.513C>G p.(Tyr171*) variant. White matter tractography shows a marked reduction of fibres within all three major fasciculi: inferior longitudinal fasciculi (ILF), superior longitudinal fasciculi (SLF) and inferior frontal-occipital fasciculi (IFOF). The colour scheme corresponds to the fibre orientation plane (green: anterior to posterior; red: left to right; blue: head to feet).

Diffusion tensor imaging tractography studies were conducted on individual NR2F1_10. Qualitative analysis of the white matter tractography data showed a striking reduction and disorganization of all three major fasciculi connected with the occipital lobe, namely, the inferior longitudinal fasciculi, the superior longitudinal fasciculi and the inferior frontal–occipital fasciculi (Fig. 3).

### Pathogenic NR2F1 variants cause significant neurodevelopmental deficits

The majority of individuals (19/22, 86.4%) presented with neurodevelopmental delay with speech and motor delays being common manifestations. Seven (31.8%) individuals were diagnosed with autistic spectrum and/or attention deficit hyperactivity disorders (Supplementary Table 1). A review of the available neuroimaging studies revealed abnormalities in 15 (79%) individuals with thinning of the optic nerve, optic chiasm and corpus callosum being frequent findings.

### Dynamic expression of NR2F1 in the developing human retina

**Figure 4:**
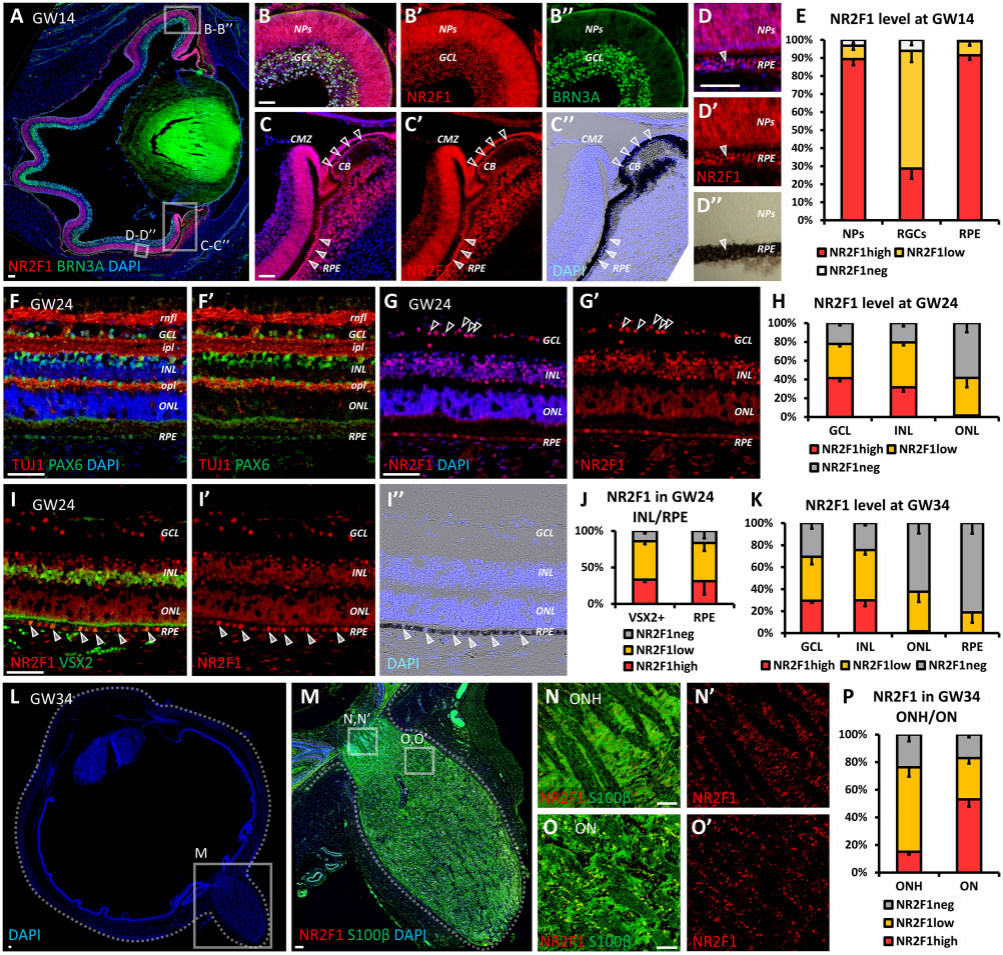
**NR2F1 expression during human retinal development.** (**A–E**) BRN3A (green; RGCs) and NR2F1 (red) immunofluorescence (IF) on a sagittal section of a human GW14 eye, showing NR2F1 expression in almost all NR progenitors and retinal ganglion cells (NPs and RGCs; B–B″), and in virtually all retinal pigmented epithelial cells (RPE; C–D″). The RPE (black) and the ciliary body (CB) are visible in merged fluorescent/brightfield (empty and full arrowheads, respectively, in C–D″). The percentage of NR2F1-expressing NPs, ganglion cell layer (GCL) cells or RPE cells is shown in graph (**E**). *n* ≥ 4/5 retinal regions from *n* = 1 eye (GW14 human embryo). (**F–H**) PAX6 (green in F, F′), NR2F1 (red in G, G′) or TUJ1 (red in F, F′) IF on GW24 NR cross-sections. Ganglion cell, inner nuclear and outer nuclear layers (GCL, INL and ONL, respectively) and retinal nerve fibre, inner plexiform and outer plexiform layers (rnfl, ipl and opl, respectively) were identified based on the position of DAPI^+^ nuclei and double staining of TUJ1/PAX6 (F, F′), which allowed the quantification of NR2F1 levels in different retinal layers (**H**). *n* = 4 retinal regions from *n* = 2 eyes (GW24 human retina). Arrowheads in (G, G′) point to cells in the GCL showing high NR2F1 level. (**I–K**) IF with VSX2 for bipolar cells or Müller glia cells (green in I) and NR2F1 (red in I, I′) on GW24 human NR. Arrowheads in (I–I″) point to RPE cells expressing high NR2F1. The proportion of VSX2+ cells or RPE cells expressing no, low or high NR2F1 is shown in graph (**J**). *n* = 4 retinal regions from *n* = 2 eyes (GW24 human retina). (**K**) Histogram showing the percentage of cells in GCL, INL, ONL and RPE expressing either low or high NR2F1 levels in GW34 human retina. *n* = 10 retinal regions from *n* = 3/4 eyes (*n* = 2 GW34 human embryos). (**L–P**) IF with S100β for astrocytes (green in M, N, O) and NR2F1 (red in M–O′) on a sagittal section of a human GW34 eye showing the optic nerve head (ONH; magnification in N, N′) and a portion of the optic nerve closest to the NR (magnification in O, O′). The proportion of ONH or optic nerve astrocytes expressing no, low or high levels of NR2F1 is shown in graph (**P**). *n* = 10 ONH/ON regions from *n* = 3/4 eyes (*n* = 2 GW34 human embryos). Nuclei (blue) were stained with DAPI. The data have been represented as mean ± SEM in the graphs. Scale bars: 50 µm, except A, L, M (100 µm). BPs, bipolar cells; CB, ciliary body; CMZ, ciliary marginal zone; GCL, ganglion cell layer; GW, gestational week; INL, inner nuclear layer; ipl, inner plexiform layer; NPs, neural progenitors; NR, neural retina; ON, optic nerve; ONH, optic nerve head; ONL, outer nuclear layer; opl, outer plexiform layer; RGCs, retinal ganglion cells; rnfl, retinal nerve fibre layer; RPE, retinal pigmented epithelium.

Staining of NR2F1 on cross-sections of human foetal eyes at gestational week (GW) 14 showed wide expression in the developing NR, with low to high levels observed in most NR progenitor cells (mean = 96.8%, SEM = 2.9%) and GCL (mean = 93.9%, SEM = 5.9%) (Fig. 4A–E). RGCs in GCL were identified with the established RGC marker Brn3a. There was also extensive NR2F1 expression in the ciliary body and in the RPE, with 99.3% (SEM = 2.5%) of cells showing low-to-high levels in the latter one (Fig. 4C–D″). RPE cells were readily recognizable by their high melanin content on brightfield microscopy (Fig. 4C″–D″). To investigate the expression of NR2F1 in the human retina at a later developmental stage, GW24 eyes were sectioned and double stained with the axonal fibre marker TUJ1, and with the PAX6 marker for RGCs and amacrine cells to distinguish the individual retinal layers (Fig. 4F–F′). Following NR2F1 immunostaining in adjacent sections, abundant signal was found in both the GCL and the INL, the latter being enriched with amacrine and bipolar cells, suggesting that NR2F1 is expressed in the majority of these three cell types (Fig. 4G–H). In comparison, 40.0% (SEM = 9.9%) of photoreceptor cells in the ONL showed weak NR2F1 expression (Fig. 4H). By taking advantage of the bipolar and Müller glia cell marker VSX2, 85.9% (SEM = 3.8%) of these cells were found to co-express low-to-high levels of NR2F1 (Fig. 4I–J). As in GW14 samples, GW24 retinas showed high NR2F1 expression in RPE cells (Fig. 4I′). NR2F1 continues to be highly expressed in the GCL and INL at GW34, but it becomes gradually downregulated in the ONL and RPE cells (Fig. 4K). In relation to the optic nerve and the optic nerve head where S100β+ astrocytes are intermingled with RGC fibres, ∼80% of S100β+ cells co-expressed low-to-high levels of NR2F1 (Fig. 4L–P). These findings demonstrate that NR2F1 is dynamically expressed in distinct retinal cell types (notably RGC, bipolar and RPE cells) during human retinal development.

### Disrupted retinogenesis in *Nr2f1* mutant mice result in non-progressive RGC loss

**Figure 5:**
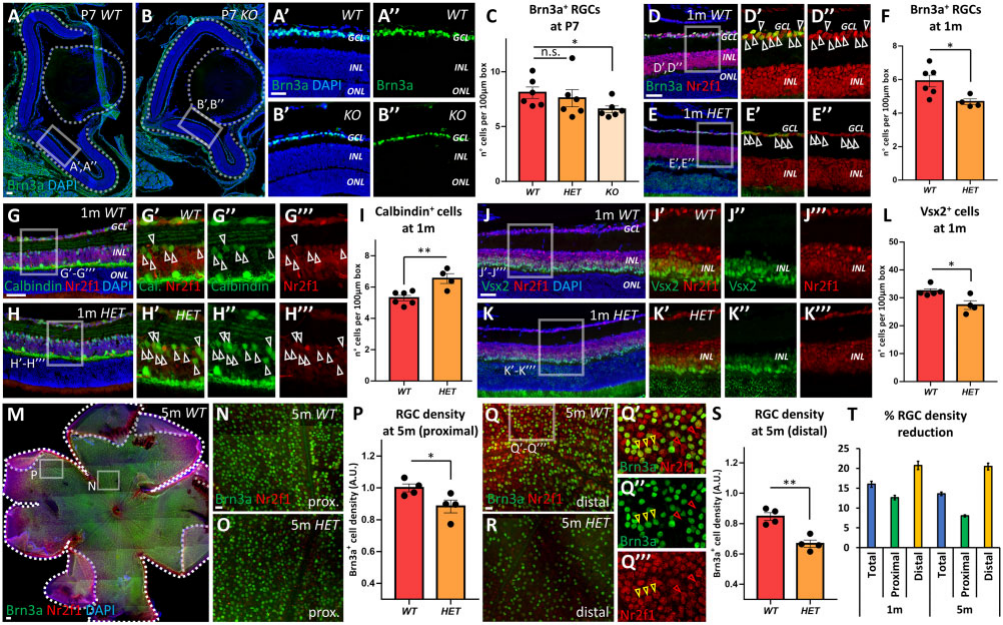
**Altered retinal development secondary to Nr2f1 loss in a mouse model.** (**A–C**) Brn3a (green; RGCs) immunofluorescence (IF) at post-natal (P) day 7 of retina from wild-type (*WT*; A–A″) and *Nr2f1* knock-out mutant (*KO*; B–B″) mice. Partial (heterozygous; *HET*) or complete (*KO*) *Nr2f1* loss leads to a reduction in Brn3a^+^ RGC count (C) [*WT*/*HET*: non-significant (n.s.) = 0.1135; *WT*/*KO*: * = 0.0325]. (**D–F**) Brn3a (green; RGCs) and Nr2f1 (red) IF on retina from one-month-old (1 m) *WT* (D–D″) and mutant *HET* (E–E″) mice. The arrowheads in (D′–E″) point to double Nr2f1^+^Brn3a^+^ RGCs. The retinal density of RGCs is quantified in (F) (*WT/HET*: * = 0.0195). For a similar quantification performed on 3-months-old retinas, see Supplementary Fig. 4C. (**G–I**) IF with Calbindin (green) for amacrine and horizontal cells, and Nr2f1 (red) in retina from 1 m-old *WT* (G–G′″) and mutant *HET* (H–H′″) mice. The relative density of Calbindin^+^ cells (ACs) in the INL is shown in (I) (*WT*/*HET*: ** = 0.0053). (**J–L**) IF with Vsx2 (green) for bipolar and Müller glia cells and Nr2f1 (red) in retina from 1 m-old *WT* (J–J′″) and mutant *HET* (K–K′″) mice. The density of Vsx2^+^ cells in INL is shown in (L) (*WT*/*HET*: *=0.0187). (**M–T**) Whole mount IF with Brn3a (green) for RGCs and Nr2f1 (red) in retina from 5-month-old (5 m) *WT* (M, N and Q–Q′″) and mutant *HET* (O, R) mice. Yellow arrowheads in (Q′–Q′″) point to double Brn3^+^Nr2f1^+^ RGC cells, while red arrowheads highlight Brn3^-^Nr2f1^+^ (probably amacrine) cells. The density of Brn3a^+^ RGCs in proximal or distal retinal regions is shown in (P) and (S), respectively, while quantification on the whole retinal surface is shown in Supplementary Fig. 4N (*WT* proximal/*HET* proximal: * = 0.05; *WT* distal/*HET* distal: ** = 0.0023). For a similar quantification performed on 1 m-old whole mount retinas, see Supplementary Fig. 4G–M. The percentages of RGC density reduction (ratio between *HET*^RGC density^ and *WT*^RGC density^) in 1 m or 5 m-old retinas and quantified in different regional regions (total, proximal and distal) are shown in Graph (T); a similar trend was found at 1 m and 5 m, suggesting relatively stable RGC reduction during mouse adult lifespan. In graphs (C, F, I, L), the number of positive cells was quantified in 60 µm-width boxes, randomly placed across the NR. In graphs (P, S), the number of Brn3a^+^ cells was counted within squares of 100 µm-width regions, randomly placed in the central (proximal) or peripheral (distal) regions of flattened whole-mount retinal explants; Brn3a^+^ cell number was normalized on the average RGC density in 5 m *WT* retinas (shown in Supplementary Fig. 4M). *n* ≥ 4/6 eyes from 2/3 animals per genotype, except P and S (*n* = 4 retinas from *n* = 2 animals per genotype). The nuclei (blue) were stained with DAPI. The data have been represented as mean ± SEM. The student’s *t*-test (F, I, L, P and S) and one-way ANOVA (C) were used for statistical analysis (*P < 0.05; **P < 0.01; ***P < 0.001). Scale bars: 50 µm, except A, M (100 µm). 1 m, 1 month-old; 3 m, 3 months-old; A.U., arbitrary unit; AC, amacrine cell; BP, bipolar cell; GCL, ganglion cell layer; INL, inner nuclear layer; NR, neural retina; ONL, outer nuclear layer; RGC, retinal ganglion cell.

The *Nr2f1* mutant mouse line was used to further investigate the role of NR2F1 in the development of the visual system. The consequences of Nr2f1 haploinsufficiency in heterozygous mutant mice (*HET*) or its complete loss in homozygous knock-out mutant mice (*KO* or *null*) were evaluated by comparing mutants with control wild-type (*WT*) littermates. The presence of ∼50% of Nr2f1 protein levels in *HET* mutants or the complete absence of Nr2f1 protein in *KO* embryos was demonstrated by immunostaining in embryonic and post-natal tissue, as well as quantified by Western blot (Supplementary Fig. 3). In this study, we further evaluated RGC numbers at later stages of retinogenesis by initially performing immunostaining of *WT* and mutant retinas with the RGC marker Brn3a at post-natal day 7 retinas (P7) (Fig. 5A–B″). *Nr2f1 KO* mice showed a significant loss of RGCs (Fig. 5A–C), whereas the RGC density in mutant *HET* mice revealed a non-significant reduction compared with control *WT* littermates (Fig. 5C). Retinas from one-month-old *WT* and *HET* animals were isolated to investigate the abundance of retinal cell populations in fully developed tissues. It was not possible to examine *KO* animals as they do not survive past P8. Brn3a-positive RGCs were significantly reduced in Nr2f1-deficient retinas (Fig. 5D–F). Notably, immunostaining performed on retinas from three-month-old *HET* animals showed a similar RGC density, indicating that no further RGC loss had occurred over time (Supplementary Fig. 4A–C). Other retinal cell populations, namely amacrine and bipolar cells, were also affected by Nr2f1-deficiency (Fig. 5G–L). There was a significant increase in Calbindin-positive amacrine cells located in mutant eyes compared with controls (Fig. 5G–I). Owing to a small expression overlap between Calbindin and Brn3a in the GCL (13.2% double positive cells; Supplementary Fig. 4D and E), only amacrine cells in the INL were included in the analysis. Vsx2-expressing bipolar and Müller glia cells were significantly reduced in *HET* mice (Fig. 5J–L), whereas the average density of photoreceptors (PRs) at one month of age was unchanged (Supplementary Fig. 4F), consistent with low expression levels of Nr2f1 in the ONL (Fig. 4H, K). Whole-mount retinal explants from one- and five-month-old animals were processed to evaluate Brn3a-positive RGC numbers and their distribution with higher spatial resolution (Supplementary Fig. 4G–N and Fig. 5M–T). The RGC density in *HET* mutants (63.7 Brn3a^+^ cells per 10.0 µm^2^, SEM = 2.6) was reduced in the proximal (central-most) retina compared with control animals (69.3 Brn3a^+^ cells per 10.0 µm^2^, SEM = 1.9, *P* = 0.044) (Fig. 5N–P). In the distal (peripheral) retina, *HET* mutants showed a more prominent reduction in RGC density (44.8 Brn3a^+^ cells per 10.0 µm^2^, SEM = 1.9) than control animals (56.3 Brn3a^+^ cells per 10.0 µm^2^, SEM = 2.4, *P* < 0.001) (Fig. 5Q–S). A comparable decrease in RGC density was observed in retinas from one- and five-month-old *HET* mutants, confirming the non-progressive nature of the RGC loss (Fig. 5T). The reduction of RGCs caused by the absence of Nr2f1 can, therefore, be detected from the early post-natal stages without further loss occurring from one month of age onwards. These observations indicate that Nr2f1 is key factor for establishing correct retinogenesis during development.

### Abnormal optic nerve development and RGC axonal guidance in Nr2f1 mutant mice

**Figure 6:**
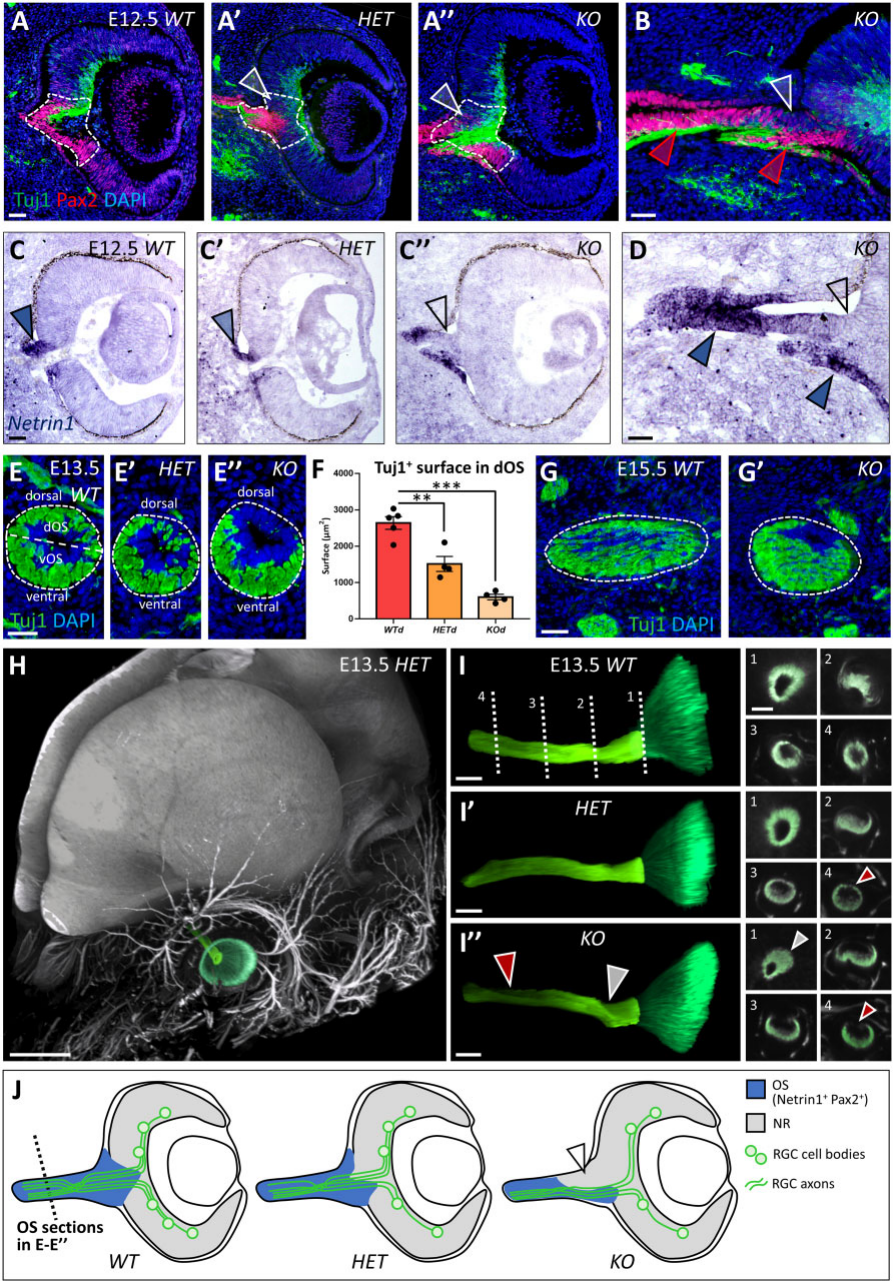
Altered optic nerve head development and Netrin1-dependent RGC axon guidance secondary to Nr2f1 loss. (**A–B**) Pax2 (red; presumptive ONH astrocytes at the NR/OS border) and Tuj1 (green; RGC axons) immunofluorescence (IF) of eye cross-sections from wild-type (*WT*; A), heterozygous (*HET*; A′) and *knock-out* mutant (*KO*; A″ and B) mice at embryonic day (E) 12.5. Partial (*HET*) or complete (*KO*) *Nr2f1* loss results in decreased number of Pax2^+^ cells located in the developing optic disc region (delineated area and white arrowheads in A–B). Red arrowheads in (B) indicate the ventral optic stalk (vOS) regions maintaining Pax2 expression. (**C–D**) *In situ* hybridization of *Netrin1* mRNA in eye cross-sections from E12.5 *WT* (C), *HET* (C′) and *KO* mutant (C″ and D) embryos. A higher magnification picture of the ONH region of a *KO* embryo is shown in (D). The dorsal optic stalk (dOS) fails to express *Netrin1* (empty arrowheads in C″ and D), whereas the vOS regions show abundant *Netrin1* expression (blue arrowheads in D). (**E–F**) Tuj1 IF showing RGC axonal fibres (green) on OS cross-sections of control *WT* (E), *HET* (E′) and *KO* mutant (E″) embryos at E13.5. Most axonal fibres follow a ventral path in *KO* OS, probably due to the absence of attractive guidance signals, such as Netrin1, in the dorsal-most ONH region. The average surface occupied by Tuj1^+^ RGC axons in dorsal OS regions is quantified in (F) (*WT/HET*: ** = <0.0013; *WT/KO*: *** = <0.0001; *HET/KO*: ** = 0.0037; *n* ≥ 4/5 optic stalks from *n* = 3 embryos per genotype). (**G–G′**) IF with Tuj1 of RGC axonal fibres (green) on OS cross-sections of control *WT* (G) and *KO* mutant (G′) embryos at E15.5, showing persisting decrease of axonal fibres in the dorsal OS of mutant animals. (**H**) Three-dimensional (3D) imaging of an E13.5 *HET* embryo head after Tuj1 IF and tissue clearing. A false green colour has been used to highlight the retina and the ON. (**I–I″**) 3D reconstruction of Tuj1^+^ axonal fibres exiting the retina (dark green) and entering the OS (light green) in *WT* (I), *HET* (I′) and *KO* (I″) E13.5 embryos. Dotted lines in (I) show the levels of transverse sections (1–4) displayed in the panels on the right. The white arrowheads point to ONH malformation in *KO* embryos, whereas the red arrowheads highlight reduced Tuj1 staining in the dorsal aspect of the developing ON in both mutant *HET* and *KO* embryos. (**J**) Schematic representation of the developing OS (blue) and NR (grey) in *WT*, *HET* and *KO* mouse eyes. As dorsal optic disc cells lose *Netrin1* expression in *KO* mutants (arrowhead), RGC axons (green) are attracted towards the ventral region of the developing ONH, where some *Netrin1* signal is still present; furthermore, RGC axons in mutants fail to invade the dorsal-most half of the developing ON. The nuclei (blue) were stained with DAPI. The data have been represented as mean ± SEM. The one-way ANOVA test was used for statistical analysis (**P < 0.01; ***P < 0.001). Scale bars: 50 µm, except H (300 µm) and G–G″ (100 µm). dOS, dorsal optic stalk; NR, neural retina; ONH, optic nerve head; OS, optic stalk; RGC, retinal ganglion cell; vOS, ventral optic stalk.

Given the prominent optic nerve head abnormalities described in individuals carrying *NR2F1* variants, optic nerve development was investigated in *HET* and *KO* mutant mice. The presumptive optic disc region, located between the NR and the OS, displayed an abnormally decreased number of Pax2-positive cells in *Nr2f1* mutants during early eye development (Fig. 6A and B). *Netrin1* expression was tested as it is known to locally guide RGC axons in the optic disc region. In keeping with an aberrant patterning at the NR/OS border, *Netrin1* was abnormally distributed in *HET* and *KO* E12.5 optic vesicles (Fig. 6C and D). Notably, *Netrin1* expression was lost in the dorsal OS of *KO* embryos (empty arrowhead in Fig. 6D), but it was maintained in the ventral OS (blue arrowheads in Fig. 6D). The early neuronal marker Tuj1 highlighted how developing RGC axons tended to be positioned ventrally in *KO* mice, presumably guided by the higher *Netrin1* ventral expression, as illustrated in transverse sections of the optic nerve (Fig. 6E and F). RGC fibres that normally fill the OS in its dorsal-most area were reduced by 48.8% (SEM = 14.8%) in *HET* and by 75.3% (SEM = 14.0%) in *KO* animals compared to control *WT* littermates (Fig. 6F). This misguidance defect was still evident at later stages (E15.5; Fig. 6G, G′, J).

Tuj1 immunostaining on clarified whole embryonic heads, followed by light-sheet imaging and 3D reconstruction, was used to visualize the RGC axonal pathway in its entirety (Fig. 6H and Supplementary Fig. 5A). At E13.5, 3D views of the retina confirmed anatomical malformations in the ventral retina and in the optic nerve head regions of mutant animals (Fig. 6I–I″ and Supplementary Fig. 5B–C′). Moreover, Tuj1^+^ fibre misguidance was readily apparent in the 3D reconstructions (Fig. 6I–I″). Virtual transverse sectioning at different levels showed that the morphological defects in mutant mice spanned the whole length of the developing optic nerve (Fig. 6I″), with RGC axons failing to fill the dorsal-most half of the optic nerve as observed in *WT* mice (Fig. 6I–I″ and Fig. 6J). Taken together, these early developmental defects emphasize the major deleterious consequences of Nr2f1 loss on the final morphology and fibre organization within the developing optic nerve, in particular the optic nerve head. A more severe defect was also observed in *KO* mice compared with *HET* mice (Fig. 6J).

### Decreased visual acuity in Nr2f1 haploinsufficient mice

To investigate whether the retinal and optic nerve defects result in functional visual impairment in the *Nr2f1* mutant mouse model, VEPs were recorded from the binocular portion of the primary visual cortex of three-month-old *WT* and *Nr2f1* haploinsufficient mice. Recordings were performed with silicon probes spanning all the cortical layers in awake, head-restrained animals (Supplementary Fig. 6A). The VEP recording has two main components, an early negative wave (N1) and a late positive peak (P1) (Supplementary Fig. 6B). The peak time of both components was significantly increased in *HET* compared with *WT* mice (Supplementary Fig. 6C and D). VEP amplitude was also significantly decreased in layers II–III of mutant animals (Supplementary Fig. 6E, G–I). The spatial resolution, which is a surrogate parameter of visual acuity, was calculated by presenting gratings of increasing spatial frequency and it was significantly reduced in *HET* mice (Supplementary Fig. 6F). Together, these data confirm impaired visual function in *Nr2f1* mutant mice, similar to what is observed in patients carrying *NR2F1* variants.

## Discussion

This study demonstrates that the optic neuropathy caused by pathogenic *NR2F1* variants is of early neurodevelopmental origin, with limited evidence of progression in later life. This is in contrast with other inherited optic neuropathies caused by pathogenic gene variants in nuclear DNA, which are typically associated with progressive visual loss from early childhood.^27^ In the current patient cohort, 22 individuals with pathogenic variants in *NR2F1* were investigated, including familial cases. Despite the high prevalence of visual system deficits in previously reported individuals with *NR2F1* variants,^9^^,^^14–18^^,^^28^ there are limited data on the ocular phenotype and the disease mechanisms that contribute to visual loss in affected individuals, since previous reports mainly focussed on the systemic clinical features.^19^^,^^29^ Furthermore, the developmental pattern and timing of NR2F1 expression in different human retinal cell types have not been assessed.

A detailed ophthalmological characterization of the *NR2F1* patient cohort points towards the visual loss in affected individuals being congenital in origin, with marked loss of neural cells within the inner retinal layers of the macula, and non-progressive in later life. Visual impairment was apparent in early childhood with nystagmus, fixation problems, strabismus and relatively preserved visual acuity compared to the structural changes within the retina. The youngest individual was diagnosed at the age of 3 months old and there was no evidence of progression in those with follow-up data, which ranged from 9 months to 17 years. Of note, a proportion of individuals were diagnosed with ONH in addition to OA, and hyperopia was the predominant refractive error. As hyperopia can be amblyogenic, early effective intervention to correct the underlying refractive error should be considered to prevent secondary vision deterioration.

The description of ONH may sometimes be confused with OA and their co-occurrence can lead to diagnostic challenges.^30^ ONH is associated with poor fixation, abnormal eye movements, nystagmus, strabismus, hyperopia,^31^ and vision ranging from no light perception to good functional vision,^32^^,^^33^ similar to the findings in this study. Moreover, ONH is often syndromic in nature occurring in conjunction with structural malformations of the brain.^7^ The most common neuroanatomical malformation found in patients with ONH is hypoplasia of the corpus callosum associated with developmental delay, neurological deficits and seizures,^6^^,^^34^ which are all clinical features observed in children carrying disease-causing NR2F1 variants.^16^^,^^19^ In addition, ONH is characterized by congenital deficiency of RGCs and their axons, which lead to disorganization of the GCL, RNFL thinning, and a small optic disc with a thin optic nerve. Various theories have been proposed to explain the aetiology of ONH, including a developmental failure of RGCs.^35–38^

Available monocular pattern reversal VEPs were all severely abnormal. A better formed or detectable binocular response suggested that the manifest/latent nystagmus was contributory to the poor monocular responses in a minority of patients. The normal or near-normal PERG P50 components (recorded binocularly and thus less susceptible to latent nystagmus) indicate preserved macular function, and the markedly abnormal PVEPs are thus not a consequence of macular disease. The PERG abnormalities included a shortened P50 peak time and reduction in the N95:P50 ratio, consistent with RGC dysfunction. Shortening of P50 peak time, possibly with some P50 component involvement, has been demonstrated clinically in various disorders including demyelination, inherited optic neuropathies and optic nerve compression,^39^^,^^40^ but also occurs following tetrodotoxin blocking of spiking cell function in a non-human primate.^41^ The P50 component is not abolished simply by loss of RGC function. Flash VEPs (mostly unaffected by nystagmus) were grossly abnormal in the majority of patients, with waveform distortions and a lack of the typical major positive components. There was no evidence from the normal ERGs obtained in the limited subgroup of patients in whom ERG was performed of any retinal dysfunction, and the abnormal flash VEPs therefore reflect post-retinal dysfunction. ERGs included assessment of the oscillatory potentials, thought to arise largely in response to activity of the amacrine cells. There was no association between the severity of VEP abnormalities and age, broadly in keeping with stable visual pathway dysfunction and the clinical data. Some patients may show slight improvement in the electrophysiological data over time, but this may simply relate to lessening of the nystagmus or improved compliance.

Both axonal damage with RNFL thinning and neuronal cell body degeneration with GCL thinning were quantified in affected individuals carrying pathogenic *NR2F1* variants by high-resolution OCT imaging. The RNFL, GCL and IPL layers, which contribute to the ganglion cell complex, were significantly thinner in the patient group compared with age-matched healthy controls. The thinning of the RNFL layer was segmental in nature compared with other inherited optic neuropathies, such as *OPA1*-related and *WFS1*-related dominant optic atrophy, in which more generalized RNFL thinning has been reported in all segments.^42–44^ It is well established that the ganglion cell complex becomes thinner in diseases affecting RGCs, including inherited optic neuropathies.^45^^,^^46^ The INL, consisting of the dendrites of RGCs, bipolar, amacrine and Müller glial cells, was significantly thicker in our *NR2F1* cohort compared with age-matched controls. Thickening of the INL has been observed in advanced glaucoma cases and a possible explanation are Müller glial cells undergoing morphological changes and hypertrophy in response to the underlying insult.^47–49^ Diffusion tensor imaging tractography imaging was available for one individual, NR2F1_10, and it is possible that defective connections of the extracortical visual pathways are contributing to visual-oriented neurodevelopmental problems such as impaired object recognition performance.^50^

Consistent with the ocular features described in BBSOAS, NR2F1 is highly expressed in the human eye, following cell differentiation from mitotic retinal progenitors to post-mitotic RGCs, as previously suggested.^19^ The genomic sequence and function of NR2F1 have been highly conserved in evolution, providing the opportunity to use mouse models to investigate the role of this gene in brain pathologies.^10^^,^^19^ It was recently demonstrated that Nr2f1-deficient mice recapitulate some of the key deficits observed in affected individuals, in particular, the ocular defects,^19^ and neocortical malformations.^20^ The *Nr2f1* mutant mouse model was therefore used to provide further insights into the relevance of NR2F1 to retinal and optic nerve development at later post-natal stages and during adulthood. *HET* mice replicate the human form of the disease, in which one allele has been lost resulting in decreased NR2F1 protein production.^19^^,^^51^ Several features of abnormal visual system development, such as ONH, early differentiation of RGCs at embryonic and early post-natal stages and cerebral visual impairment, could be faithfully recapitulated and investigated using this well-characterized mouse model.^19^ As RGCs elongate their axons to form the optic nerve, the reduced RGC density with thinning of the GCL offers an obvious explanation for the development of ONH in this mouse model (this study and reported by Bertacchi et al.^19^). Other retinal cell populations, in particular, Calbindin-expressing amacrine and Vsx2-expressing bipolar and Müller glia cells, are also affected by Nr2f1 haploinsufficiency, suggesting that RGC layer imbalance secondary to Nr2f1 loss could have deleterious consequences for the retinal circuitry as a whole.^52^ However, the marked loss of RGCs remains the primary characteristic of *Nr2f1* haploinsufficient mice, detectable during early post-natal development and confirmed in one-, three- and five-month-old retinas with no progressive degeneration. This matches the findings in our *NR2F1* patient cohort with OCT imaging showing RGC layer thinning and visual electrophysiology confirming RGC dysfunction. Of note, RGC density remained stable in post-natal and adult Nr2f1 mutant mice. Given that affected individuals in the *NR2F1* patient cohort retained relatively stable visual acuities during an extended period of follow-up, the overall evidence points towards a non-progressive process mainly due to a failure of RGC production in early embryonic development, rather than a more gradual loss of RGCs over time. This explanation would also account for the high prevalence of ONH in this patient population.

Nr2f1 has been proposed to orchestrate the expression of key molecular determinants, such as Pax6 and Pax2, in the NR and OS, including the border regions corresponding to the developing optic nerve head.^19^^,^^53^ This structure constitutes a critical region for eye function with secreted molecules, such as *Netrin1*, locally guiding RGC axons as they exit the retina and promoting their survival during neuronal navigation.^54–56^ Indeed, lack or reduced *Netrin1* expression prevents the organized topographical migration of RGC axons from the NR into the OS, causing optic disc abnormalities if the dysfunction is severe enough.^54^^,^^55^ In this study, we define an important pathological mechanism arising from the loss of Nr2f1. Strikingly, the optic disc lacks a properly defined key molecular signature (Netrin1) that normally guides RGC axons from the inner retina towards the OS, resulting in misrouting of RGC fibres. By employing novel tissue clarification protocols,^57^^,^^58^ we were able to generate a detailed structural map of the axonal fibres exiting the NR and entering the optic disc. The morphological defects spanned the whole length of the OS and as one would expect from the complete loss of Nr2f1, the failure of proper RGC axonal navigation was more pronounced in *KO* mice. Previous studies in mice have demonstrated a role for Nr2f1 in regulating axonal elongation and guidance in different brain regions.^26^ The present data suggest a similar role in the visual system, both at the level of the retina by orchestrating *Netrin1* expression and RGC differentiation, and at the central brain tract level, as demonstrated by white matter tractography imaging. Altogether, the data indicate that loss of *Nr2f1* expression affects the organization of the optic nerve head region, further reinforcing the notion that visual loss in individuals carrying pathogenic *NR2F1* variants arise from pathological events in early development. Furthermore, given the highly conserved evolutionary role of NR2F1,^29^ we hypothesize that the molecular domain shift at the optic nerve head border could account for the ONH described in a proportion of affected individuals.

Finally, electrophysiological recordings were used to evaluate visual system spatial resolution, a surrogate of visual acuity, in *Nr2f1* mutant mice. *Nr2f1* haploinsufficiency led to delayed signal transmission and reduced VEP amplitude, in keeping with the observed optic nerve abnormalities and the retinal alterations in bipolar and RGC cell populations. Spatial resolution (acuity) was also significantly decreased. The *Nr2f1 HET* mouse may, therefore, represent an efficient system to recapitulate the vision loss in BBSOAS and, more generally, an attractive genetic model to further study retinogenesis and the mechanisms driving RGC loss in early development.

In conclusion, this study provides a comprehensive description of the ophthalmological phenotypes in a cohort of individuals carrying pathogenic *NR2F1* variants, providing new insights into the underlying structural ocular defects and functional consequences on vision. The findings from the *Nr2f1* mouse model indicate that the retinal and optic nerve head anomalies associated with Nr2f1 deficiency represent a primary RGC patterning and axonal guidance defect originating during early development. These observations are particularly relevant given that half of all patients carrying pathogenic *NR2F1* variants develop ONH as a prominent ocular feature. Furthermore, the longitudinal visual data collected in our NR2F1 patient cohort indicate that the visual loss arises due to an insult occurring early in development with no evidence of marked progression later in life. A prospective study involving a larger cohort of patients assessed using a standardized protocol and spanning a longer follow-up period is needed to confirm that visual loss is non-progressive in BBSOAS, which has important implications for genetic counselling and patient management.

## Supplementary material

Supplementary material is available at *Brain Communications* online.

The authors would like to thank Dr Cara M. Skraban (Roberts Individualized Medical Genetics Center, Children’s Hospital of Philadelphia, Philadelphia, PA, USA) for assistance with clinical characterization and genetic testing. We are also grateful to Dr Fabien Guimiot from the Inserm U1141 Unit at the Robert Debré Hospital (Paris, France) for providing us with GW24 and GW34 human eye samples, and to Ms C. Allet and Dr P. Giacobini (Lille, France) for the GW14 cryostat sections. Finally, we also thank the iBV animal facility and K. Moneret for animal handling and care.

## Funding

N.J. was supported by the Moorfields Eye Charity (GR001203), National Eye Research Centre (SAC051), National Institute of Health Research Biomedical Research Centre at Moorfields Eye Hospital and UCL Institute of Ophthalmology. P.Y.W.M. was supported by a Clinician Scientist Fellowship Award (G1002570) from the Medical Research Council (UK), and also receives funding from Fight for Sight (UK), the Isaac Newton Trust (UK), the Addenbrooke’s Charitable Trust, the National Eye Research Centre (UK), the International Foundation for Optic Nerve Disease, the UK National Institute of Health Research as part of the Rare Diseases Translational Research Collaboration, the National Institute of Health Research Cambridge Biomedical Research Centre (BRC-1215-20014), and the National Institute of Health Research Biomedical Research Centre based at Moorfields Eye Hospital NHS Foundation Trust and UCL Institute of Ophthalmology. G.A. was supported by a Fight for Sight (UK) Early career Investigator Award, the National Institute of Health Research Biomedical Research Centre based at Moorfields Eye Hospital NHS Foundation Trust and UCL Institute of Ophthalmology and the National Institute of Health Research Biomedical Research Centre at Great Ormond Street Hospital Institute for Child Health. A.G.R. was supported by the National Institute of Health Research Biomedical Research Centre based at Moorfields Eye Hospital NHS Foundation Trust and UCL Institute of Ophthalmology and by the Moorfields Eye Charity. G.E.H. was supported by the National Institute of Health Research Biomedical Research Centre based at Moorfields Eye Hospital NHS Foundation Trust and UCL Institute of Ophthalmology, and The Foundation Fighting Blindness (USA). N.P. was supported by a Moorfields Eye Charity Career Development Award (R190031A). The views expressed are those of the author(s) and not necessarily those of the NHS, the National Institute of Health Research or the Department of Health. The next generation sequencing in individuals from South Korea was supported by a fund (#2018-ER6902-00) from the Research of Korea Centers for Disease Control and Prevention. This work was supported by European Research Area Networks Neuron II grant (Improv-Vision) ANR-15-NEUR-0002-04, by the Jerome Lejeune Foundation (grant N° 199162) and by ‘Investments for the Future’ LabEx SIGNALIFE (grant ANR-11-LABX-0028-01) to M.S.

## Competing interest

The authors report no competing interests.

## Appendix

The Genomics England Research Consortium:

J.C. Ambrose, P. Arumugam, R. Bevers, M. Bleda, F. Boardman-Pretty, C.R. Boustred, H. Brittain, M.J. Caulfield, G.C. Chan, T. Fowler, A. Giess, A. Hamblin, S. Henderson, T.J.P. Hubbard, R. Jackson, L.J. Jones, D. Kasperaviciute, M. Kayikci, A. Kousathanas, L. Lahnstein, S.E.A. Leigh, I.U.S. Leong, F.J. Lopez, F. Maleady-Crowe, M. McEntagart, F. Minneci, L. Moutsianas, M. Mueller, N. Murugaesu, A.C. Need, P. O’Donovan, C.A. Odhams, C. Patch, D. Perez-Gil, M.B. Pereira, J. Pullinger, T. Rahim, A. Rendon, T. Rogers, K. Savage, K. Sawant, R.H. Scott, A. Siddiq, A. Sieghart, S.C. Smith, A. Sosinsky, A. Stuckey, M. Tanguy, A.L. Taylor Tavares, E.R.A. Thomas, S.R. Thompson, A. Tucci, M.J. Welland, E. Williams, K. Witkowska, S.M. Wood.

## Supplementary Material

fcab162_Supplementary_Data

## Abbreviations

BBSOAS =: Bosch–Boonstra–Schaaf Optic Atrophy Syndrome
BCVA =: best-corrected visual acuity
DBD =: DNA-binding domain
ERG =: full-field electroretinography
FVEP =: flash visual evoked potential
GCL =: retinal ganglion cell layer
GW =: gestational week
HET =: heterozygous
IF =: immunofluorescence
INL =: inner nuclear layer
IO =: inferior outer
IPL =: inner plexiform layer
ISCEV =: International Society for Clinical Electrophysiology of Vision
KO =: homozygous
LBD =: ligand-binding domain
NP =: neural progenitors
NR =: neural retina
OA =: optic atrophy
OCT =: optical coherence tomography
ONH =: optic nerve hypoplasia
ONL =: outer nuclear layer
OS =: optic stalk
PERG =: pattern electroretinography
PVEP =: pattern visual evoked potentials
RGC =: retinal ganglion cell
RNFL =: retinal nerve fibre layer
RPE =: retinal pigment epithelium
SEM =: standard error of the mean
SO =: superior outer
VEP =: visual evoked potentials
