# Supplementary material for: Pathogenic NR2F1 variants cause a developmental ocular phenotype recapitulated in a mutant mouse model
Source: Brain Commun. 2021 Jul 20;3(3):fcab162. doi: 10.1093/braincomms/fcab162 (PMC8397830; doi:10.1093/braincomms/fcab162)
Supplement: fcab162_Supplementary_Data [file fcab162_supplementary_data.zip › Supplementary_material_GERC_author_list.pdf]

# Genomics England Research Consortium

## The Genomics England Research Consortium

Ambrose, J. C. [1]; Arumugam, P.[1]; Bevers, R.[1]; Bleda, M.[1]; Boardman-Pretty, F.[1,2]; Boustred, C. R.[1]; Brittain, H.[1]; Caulfield, M. J.[1,2]; Chan, G. C.[1]; Fowler, T.[1]; Giess A. [1]; Hamblin, A.[1]; Henderson, S.[1,2]; Hubbard, T. J. P.[1]; Jackson, R. [1]; Jones, L. J. [1,2]; Kasperaviciute, D.[1,2]; Kayikci, M.[1]; Kousathanas, A. [1]; Lahnstein, L.[1]; Leigh, S. E. A.[1]; Leong, I. U. S.[1]; Lopez, F. J.[1]; Maleady-Crowe, F.[1]; McEntagart, M.[1]; Minneci F. [1]; Moutsianas, L.[1,2]; Mueller, M.[1,2]; Murugaesu, N. [1]; Need, A. C.[1,2]; O'Donovan P.[1]; Odhams, C. A.[1]; Patch, C.[1,2]; Perez-Gil, D.[1]; Pereira, M. B.[1]; Pullinger, J.[1]; Rahim, T.[1]; Rendon, A.[1]; Rogers, T.[1]; Savage, K.[1]; Sawant, K. [1]; Scott, R. H.[1]; Siddiq, A.[1]; Sieghart, A.[1]; Smith, S. C. [1]; Sosinsky, A.[1,2]; Stuckey, A.[1]; Tanguy M. [1]; Taylor Tavares, A. L.[1]; Thomas, E. R. A.[1,2]; Thompson, S. R.[1]; Tucci, A.[1,2]; Welland, M. J.[1]; Williams, E.[1]; Witkowska, K.[1,2]; Wood, S. M.[1,2].

1. Genomics England, London, UK

2. William Harvey Research Institute, Queen Mary University of London, London, EC1M 6BQ, UK.

27th May 2021
