# Supplementary material for: Pathogenic NR2F1 variants cause a developmental ocular phenotype recapitulated in a mutant mouse model
Source: Brain Commun. 2021 Jul 20;3(3):fcab162. doi: 10.1093/braincomms/fcab162 (PMC8397830; doi:10.1093/braincomms/fcab162)
Supplement: fcab162_Supplementary_Data [file fcab162_supplementary_data.zip › Supplementary_material.pdf]

Protocols for immunofluorescence (IF), Western blot (WB), in situ hybridisation (ISH), intracortical mouse visual evoked potential (VEP) recording, and three-dimensional (3D) imaging of mouse tissues

Immunofluorescence (IF)

Mouse embryonic retinas and whole heads were dissected and fixed in 4% paraformaldehyde (PFA) at 4°C for 3 hours in agitation, then washed in PBS 1X and dehydrated in 25% sucrose overnight at 4°C. For paraffin-embedded sections (GW24 and GW34 human retinas), the initial washes consisted in 3 washes in Xylene (5' each), followed by 2 washes in 100% Ethanol (10' Each) and gradual rehydration (80%, 60% and 30% Ethanol 10' washes followed by 2X5' washes in milliQ-H_2O). All antibodies required antigen retrieval prior to incubation (10 minutes at 95°C in pH=6 Citric acid solution). Primary antibodies used were: NR2F1 (Abcam ab181137, 1:1000, rabbit; R&D H8132, 1:1000, mouse); Brn3a (1:1000, mouse, kind gift of Thomas Lamonerie, iBV, Nice); Pax6 (Millipore AB2237; 1:500, rabbit); Vsx2 (Exalpha X1180P, 1:200, sheep); S100β (Dako Cytomation Z0311, 1:200, rabbit); Tuj1 (β-III Tubulin, Sigma T8660, 1:1000, mouse); Calbindin (Swant CB-38a, 1:500, rabbit). Alexa Fluor 488, 555, 594 and 647 anti-mouse, anti-rabbit or anti-sheep IgG conjugates (Thermo Fisher scientific, all 1:500) were used as secondary antibodies. Images were acquired at an Apotome Zeiss, using the AxioVision software.

Western blotting (WB)

Embryonic day 12.5 embryos were sacrificed by decapitation and heads were transferred in ice-cold PBS1x immediately after. Heads were lysed in RIPA buffer (10 mM Tris-Cl (pH 8.0), 1 mM EDTA, 1% Triton X-100, 0.1% sodium Deoxycholate, 0.1% SDS, 140 mM NaCl, 1 mM PMSF) implemented with Complete protease inhibitors (Roche); three 5 minutes intervals at 95°C were alternated with tissue disruption by pipetting through a small insulin syringe needle. Protein quantity was then measured with the Pierce BCA protein assay KIT (ThermoScientific). After quantification, 1 volume of NuPAGE buffer with 5% β-Mercaptoethanol was added for 3 sample volumes, then samples were boiled 5 minutes at 95°C for denaturation. Equal amounts of proteins from tissue lysates (10 μg per sample) were resolved by reducing SDS-PAGE (12% Acrylamide gel), transferred to PolyVinylidene DiFluoride (PVDF) membrane and incubated with the desired antibodies (NR2F1, Abcam ab181137, 1:4000, rabbit; β-Actin; Abcam ab8277, 1:5000, rabbit). Blocking was performed with 5% BSA (or 5% milk) in Tris-Buffered Saline (TBS), while washes were done in TBST

(TBS + 0.1% Tween). Immunoblots were developed using ECL Prime western blotting detection reagent (GE Healthcare, RPN2232). After Nr2f1 immunoblotting, the membrane was treated with 10 ml of Restore Western Blot Stripping Buffer (Thermo scientific REF 21059; 15 minutes on shaker at RT), then used for β-Actin immunoblotting. For quantification, ImageJ was used; Nr2f1 sample signals were normalized on the signal intensity of the housekeeping protein β-Actin.

In situ hybridisation **(ISH)**

Netrin1 RNA probes were transcribed *in vitro* from a previously used plasmid (Deiner *et al.*, 1997). Embryos for ISH were fixed overnight with PFA 4% at 4°C, dehydrated in 25% sucrose, and embedded in OCT resin to be stored at -80°C and cut at cryostat (Leica; 14μm cryosections). Defrosted and air-dried head sections were washed twice in PBS 1X then treated with RIPA buffer (150nM NaCl, 1% NP-40, 0.5% Na deoxycholate, 0.1% SDS, 1mM EDTA, 50mMTris [pH 8.0]) for 10 min. Pre-hybridization and hybridization of the sections were performed in the following solution: 50% formamide, 5X saline sodium citrate (SSC), 5X Denhardt's solution (Invitrogen), 500 μg/ml salmon sperm DNA (Ambion), 250 μg/ml yeast tRNA, *Netrin1* RNA probe 1000 ng/ml. Hybridization step was performed O/N at 65°C. After washes at 65°C in 50% formamide, 2X SSC, 0.1% Tween 20, the samples were equilibrated in MABT (maleic acid buffer containing 0.1% Tween 20) solution and blocked in MABT/10% sheep serum. The hybridized probes were detected by overnight incubation at 4°C with an anti-DIG antibody (ENZO ENZ-ABS266-0100, 1:2000). Finally, sections were washed several times in MABT, then equilibrated in B3 buffer (100 mM Tris [pH 9.5], 50 mM $MgCl_2$, 100 mM NaCl, 0.1% Tween 20). The staining was performed by incubating the slices in NBT/BCIP solution (Roche) at room temperature (or overnight at 4°C). Two quick washes with B3 buffer supplemented with tetramisole (0.5 mg/mL; Sigma) were performed before staining to reduce background.

Tissue clearing and 3D imaging

Embryos were collected at E13.5, and heads briefly rinsed in PBS prior incubation in PFA 4% for 3-4h at 4°C, with gentle shaking. Fixed tissues were then processed according to previously published protocols (Belle *et al.*, 2017; Henning *et al.*, 2019) with some modifications. To quench tissue auto-fluorescence and decolour the retina pigmented layer, heads were treated with 10% H_2O_2 in PBS, for 1h 30' at 55°C. Then, embryo heads were incubated in PBSGT blocking buffer (0.5% Triton-X 100, 0.2% Gelatin in PBS-Azide)

overnight (O/N) at 37°C with gentle shaking or slow rotation. Heads were then incubated with the Tuj1 primary antibody (BioLegend #802001; 1:500 in PBSGT) for 5 days at 37°C in slow rotation. After incubation, samples were repeatedly washed in PBST (0.5% Triton-X 100 in PBS) over the day at room temperature (RT) in slow rotation, then O/N at 4°C. Secondary antibody (Thermo Fisher scientific; anti-rabbit Alexa-fluor 555; 1:500 in PBSGT) was added and heads were incubated O/N at 37°C in slow rotation. After several washes in PBST (last wash O/N at 4°C), heads were dehydrated in increasing concentration of methanol in PBS pH 7.4 (20%; 40%; 60%; 80% and finally twice in 100%; 1h each step, RT in slow rotation; quicker rotation is recommended for 100% methanol washes. Lipid removal was obtained with 66% dichloromethane (DCM) in methanol by O/N incubation at RT, followed by 1h-long incubation in 100% DCM at RT in slow rotation. Finally, refractive index matching and tissue clearing were obtained by submerging the samples in dibenzil-ether (DBE). Tissues were stored at RT and in the dark until imaging. Images were acquired *via* the Zeiss light-sheet Z.1 microscope equipped with 5x objective and Zen software. Image analysis, 3D rendering and signal segmentation, as well as video editing were performed using the IMARIS (Bitplane) software.

Mouse electrophysiology for Visual-Evoked Local Field Potential Recording

Surgery Procedure and Animal Preparation. Mice were deeply anesthetized with an intraperitoneal injection of avertin (0.020 ml/g), and positioned on a stereotaxic frame; the scalp was partially removed, the skull cleaned and dried. A ground screw was implanted above the cerebellum. Mice were implanted with a custom-made lightweight head post, placed on the skull on the left hemisphere, aligned with the sagittal suture and cemented in place with a dental adhesive system (Super-Bond C&B). A thin layer of the dental cement was used to cover the entire exposed skull. A recording chamber was built using a dental cement (resin adhesive cement, Ivoclar Vivadent) and centered on binocular visual cortex (2 x 2 mm, ~ 3 mm medio lateral to Lambda). The skull over the recording area was covered by sterile low melting agarose Type III (A6138, Sigma-Aldrich, Inc.) and sealed with Kwik-Cast (WPI). Mice were allowed to awaken and then housed separately.

Visual evoked potential recording. The electrophysiological data were continuously bandpass filtered (0.5 Hz to 6 kHz), using a 16-channel Omniplex recording system (Plexon, Dallas, TX). A NeuroNexus Technologies 16-channel linear silicon probe with a single-shank (A1x16-3mm-50-177, 50μm spacing among contacts) was slowly lowered into the visual

cortex; the tip of the probe was placed at about 800 µm depth using a fine micromanipulator. The recording chamber was filled with sterile saline solution (NaCl 0.9%). The electrode was allowed to stabilise for approximately 10 minutes prior to commencing recording. One to 3 extracellular recording sessions were performed for each animal.

Visual stimuli. Visual stimuli were computer-generated using the Matlab Psychophysics Toolbox with gamma correction on a display (Sony; 40 x 30 cm; mean luminance 15 cd/m^2) placed about 25 cm from the mouse, covering the center of the visual field. Transient VEPs were recorded in response to abrupt reversal (1 Hz) of a horizontal square wave grating (spatial frequency 0.05, 0.06, 0.2, 0.3, 0.4, 0.5 and 0.6 c/deg; contrast 30%). At least 30 events were averaged in synchrony with the stimulus contrast reversal. The response to a homogeneous grey screen (0% contrast, K0) was also frequently recorded to estimate noise.

Neurophysiological data pre-processing. All the electrophysiological data were processed blind to animal treatment using custom-written MATLAB code (The MathWorks, Natick, MA, USA). Transient VEPs were evaluated in the time domain by measuring the peak-to-trough amplitude and peak times of the major negative (N1) and positive (P1) components. Visual acuity was measured as the highest spatial frequency that evoked a response above noise level at 30% contrast.

Supplementary Table 1. Summary of systemic manifestations in the *NR2F1* patient cohort.

Subject	Neurodevelopmental deficits and learning disability	Balance and coordination deficits	Epilepsy/seizures	Other clinical features	Neuroimaging findings*
NR2F1_1	Developmental delay (mild), but almost normal speech abilities. Behavioural disorders: attention deficit hyperactivity disorder and autism spectrum disorder.	Yes	Occasional generalized epileptic-like anomalies during light sleep. Febrile seizures (4 and 8 years of age).	Low thyroid-stimulating hormone. Hypotonia and hyperlaxity. Mild facial asymmetry.	Thinning of corpus callosum
NR2F1_2	Developmental delay and speech difficulties. Learning disability.	Yes	No	Mild facial dysmorphism	Normal
NR2F1_3	Developmental delay: walking at 15 months of age. Learning disability.	No	No	No	Normal
NR2F1_4	Developmental delay and speech difficulties. Precocious puberty. Behavioural disorders: stereotypical movements/repetitive behaviour; attention deficits. Learning disability.	No	Epileptic seizures (3-4 episodes per year)	Hypotonia	Thinning of the posterior half of the corpus callosum. Thinning of the optic chiasm and superior cerebellar vermis atrophy. Abnormal gyral pattern involving the left supramarginal and angular gyri.
NR2F1_5	Global developmental delay and delayed visual maturation. Autism spectrum disorder. Learning disability.	Yes	Febrile seizure (single episode)	Preterm birth (gestational age of 34 weeks and 6 days). Macrocephaly. Morgagni hernia (laparoscopic repair). Arterial wall aneurysm. History of elevated liver enzymes. History of respiratory distress. Moderate-severe hypotonia.	Moderate diffuse thinning of the posterior body and splenium of the corpus callosum. Mild T2 prolongation in the periatrial region with associated white matter volume loss (periventricular leukomalacia). Diffuse marked thinning of the optic chiasm and optic nerves bilaterally with abnormal T2/FLAIR hyperintensity.
NR2F1_6	Global developmental delay and learning disability.	No	No	Ocular torticollis. Hypotonia.	Minimally foreshortened corpus callosum. Slightly hyperintense central tegmental tracts and brainstem (nonspecific).
NR2F1_7	Pervasive developmental disorder with speech delay and global apraxia. Behavioural disorders: attention deficit hyperactivity disorder and autism spectrum disorder. Learning disability.	Yes	Myoclonic epilepsy. Focal impaired-awareness seizures.	Recurrent otitis media	Normal except for subtle white matter abnormalities in the inferior optic radiations and occipital lobes.
NR2F1_8	Global developmental delay. Learning disability.	Yes	Infantile spasms. Myoclonic epilepsy.	Mild dysmorphism. Hypotonia.	Decreased white matter volume, most notably at the level of the centrum semiovale consistent with atrophy. Thinning of the corpus callosum with decreased myelination, in particular at the level of the splenium.
NR2F1_9	Developmental delay: delayed fine and gross motor skills, developmental coordination disorder, expressive language delay and dyslexia. Autism spectrum disorder with anxiety and limited attention span. Learning disability.	Yes	No	Silent gastro-esophageal reflux in infancy. Frequent ear infections resolved with grommets.	General reduction in white matter volume with slender optic nerves
NR2F1_10	Developmental delay. Attention deficit hyperactivity disorder. Learning disability.	Yes	No	Mild facial dysmorphism including retrognathia and external ear protrusion.	Thinning of the posterior portion of the corpus callosum

NR2F1_11	Global developmental delay and learning disability.	Yes	No	Congenital heart disease: atrial septal defect and patent ductus arteriosus (surgical repair). Chronic lung disease with pulmonary hypertension (bilateral upper pulmonary vein atresia). Congenital bilateral foot deformities: right vertical talus (surgical repair) and mild left hindfoot valgus deformity. Left cryptorchidism (surgical repair). Severe preterm birth (gestational age 27 weeks and 3 days). Hypotonia. Microcephaly. Dysmorphisms. Feeding difficulties and poor growth with G-tube dependence. Non-identical twin has evidence of developmental delay with complications of prematurity.	CT: Mildly prominent lateral and third ventricles. Prominence of the cerebrospinal fluid spaces over the frontal lobes. Mild prominence of the supratentorial subarachnoid spaces and ventricles, suggesting volume loss.<br><br>MRI: Borderline or low-normal thickness of the optic chiasm. Optic nerves likely still within the normal range. Olfactory bulbs not visualised on either side. Shallow or absent olfactory sulci in some parts along their course. Small subcortical 3 mm focus of increased T2/FLAIR hyperintensity and T1 hypointensity without contrast enhancement in the right posterior medial temporal lobe (unchanged on 3-month follow up MRI). Nonspecific – though possibly a small cystic structure versus low-grade neoplasm). Normal size, shape and configuration of the ventricular system.
NR2F1_12	Developmental delay: walking at 2.5 years, speech and language delay with markedly delayed expressive language skills. Limited concentration and short attention span.	Heel-to-toe walking	No	Congenital heart disease: bicuspid aortic valve. Left lacrimal duct stenosis. Recurrent ear infections with left conductive hearing impairment. Microphthalmia. Asymmetric head and alopecia on the crown. Increased tone left ankle. Sister has craniofacial abnormalities (genetically not tested).	Underdeveloped optic nerves (hypoplasia), chiasm and corpus callosum. Absent septum pellucidum and small pituitary gland in keeping with septo-optic dysplasia. Delayed maturation of the white matter for the individual's age.
NR2F1_13	Normal development	No	No		Atrophy of the optic chiasm
NR2F1_14	Developmental delay: sitting (15 months), crawling (15-16 months), walking (21 months) and speech (34 months). Dyslexia. Learning disability.	Yes	No	Frequent ear infections. Cleft palate (surgical repair). Suspected Pierre-Robin sequenced (17q24.3-q25.1 excluded by interrogating WGS data). Asthma. Joint hypermobility syndrome. Periventricular leukodystrophy from premature birth.	NA
NR2F1_15	Developmental delay: walking and speech.	Yes	No	Mild dysmorphism	NA
NR2F1_16	Developmental delay: walking and speech.	Yes	Febrile seizure (single episode)	Mild dysmorphism	Normal
NR2F1_17	Developmental delay: walking and speech.		No	Mild dysmorphism	Normal
NR2F1_18	Normal development	No	No		NA
NR2F1_19	Normal development	No	No		Atrophy of the optic nerves (slender)
NR2F1_20	Developmental delay: motor, walking and speech. Learning disability.	Yes	No	Generalized hypotonia	Slender optic nerves and global lack of white matter
NR2F1_21	Developmental delay. Autism spectrum disorder. Learning disability.	Yes	No	Motor dyspraxia	Atrophy of the optic nerves (slender) and optic chiasm
NR2F1_22	Developmental delay: mild speech and mild cognitive delay. Learning disability.	No	No		Small optic nerves, optic chiasm and optic tracts bilaterally, in keeping with the patient's optic atrophy. Nonspecific thickening of the genu, rostrum, and anterior body of the corpus callosum. Duplicated anterior communicating artery and three A2 segments of the anterior cerebral arteries noted.

* Based on the review of magnetic resonance imaging (MRI) and/or computer tomography (CT) scans. NA – not available.

Supplementary Table 2. Long-term follow up visual acuity.

Subject	Follow up duration (approximate)	Age at the examination	BCVA (logMAR)	
			RE	LE
NR2F1_5	16 months	3 months	ND[a]	ND[a]
		18 months	1.43	1.7
NR2F1_6	28 months	33 months	0.81	0.81
		5 years	0.7	0.7
NR2F1_8	3 years	9 months	1.37	1.37
		4 years	0.81	0.81
NR2F1_10	23 months	16 months	ND[b]	ND[b]
		3 years	ND[b]	ND[b]
NR2F1_7	6 years	14 years	0.1	0.18
		20 years	0	0.1
NR2F1_13	3 years	7 years	0.8	0.9
		10 years	0.6	0.9
NR2F1_14	14 years	15 years	0.46	1
		29 years	0.43	0.43
NR2F1_16	17 years	8 years	0.43	0.3
		26 years	1.48	1.48
NR2F1_17	15 years	5 years	0.92	0.92
		20 years	0.78	1
NR2F1_18	15 years	21 years	0.22	0.22
		36 years	0.22	0.22
NR2F1_19	10 years	8 years	0.6	0.8
		18 years	0.3	0.48
NR2F1_22	47 months	3 years	ND[b]	ND[b]
		7 years	1	0.88

ND – no data

a – BCVA not quantified. Patient reacted to light, no reliable fixation/follow.

b – BCVA not quantified. Patient was able to maintain fixation and follow.

Supplementary table 3. Comparison of retinal layer thickness in individuals with *NR2F1* variants and age-matched healthy controls.

Retinal layer	Circle	Segment	Layer thickness (μm) (mean ± SEM) NR2F1 (N=7 eyes)	Controls (N=7 eyes)	P-value
Full retina	Inner	Superior	327.14 ± 4.703	342.71 ± 3.428	0.026*
		Temporal	315.00 ± 14.609	326.29 ± 4.016	0.805
		Inferior	328.00 ± 4.22	338.29 ± 4.167	0.038*
		Nasal	331.43 ± 5.322	333.14 ± 4.306	0.620
	Outer	Superior	303.00 ± 2.743	340.43 ± 2.277	0.001***
		Temporal	310.43 ± 2.724	327.43 ± 3.199	0.001***
		Inferior	296.29 ± 3.099	333.29 ± 5.537	0.002**
		Nasal	315.00 ± 5.827	347.00 ± 2.828	0.001***
RNFL	Inner	Superior	17.00 ± 0.900	19.29 ± 0.522	0.073
		Temporal	17.29 ± 0.778	16.71 ± 0.837	0.620
		Inferior	18.00 ± 0.69	19.29 ± 0.944	0.456
		Nasal	17.43 ± 0.997	17.57 ± 0.297	0.318
	Outer	Superior	19.00 ± 0.900	31.71 ± 1.209	0.001***
		Temporal	18.86 ± 0.769	17.86 ± 0.670	0.383
		Inferior	19.86 ± 1.204	31.29 ± 2.157	0.004**
		Nasal	19.14 ± 1.056	26.14 ± 0.986	0.001***
GCL	Inner	Superior	36.00 ± 2.734	54.43 ± 0.719	0.001***
		Temporal	34.00 ± 3.612	43.00 ± 1.234	0.073
		Inferior	38.86 ± 2.521	51.43 ± 1.716	0.002**
		Nasal	33.57 ± 2.999	46.00 ± 1.195	0.004**
	Outer	Superior	34.71 ± 1.063	51.71 ± 0.993	0.001***
		Temporal	32.86 ± 2.064	50.86 ± 1.710	0.001***
		Inferior	35.00 ± 0.756	51.00 ± 2.093	0.001***
		Nasal	37.86 ± 2.613	57.57 ± 0.719	0.001***
IPL	Inner	Superior	35.71 ± 1.267	42.43 ± 0.649	0.001***
		Temporal	39.57 ± 1.757	39.43 ± 0.841	0.456
		Inferior	37.00 ± 0.690	41.43 ± 0.896	0.004**
		Nasal	38.86 ± 0.986	40.00 ± 0.787	0.620
	Outer	Superior	30.57 ± 0.751	40.29 ± 0.918	0.001***
		Temporal	35.43 ± 1.307	42.86 ± 1.223	0.001***
		Inferior	29.71 ± 0.421	39.57 ± 1.494	0.001***
		Nasal	34.43 ± 1.702	43.86 ± 0.738	0.001***
INL	Inner	Superior	46.86 ± 1.438	39.57 ± 1.395	0.007**
		Temporal	42.29 ± 1.614	36.00 ± 1.676	0.026*
		Inferior	46.71 ± 1.459	40.71 ± 1.599	0.026*
		Nasal	45.57 ± 2.698	36.86 ± 1.079	0.002**
	Outer	Superior	42.71 ± 0.808	42.29 ± 1.040	0.805
		Temporal	44.29 ± 0.680	39.86 ± 0.962	0.007**
		Inferior	41.57 ± 1.152	42.57 ± 1.913	0.805
		Nasal	45.86 ± 1.710	42.43 ± 0.812	0.053
ONL	Inner	Superior	71.57 ± 4.117	61.29 ± 5.891	0.259
		Temporal	76.86 ± 6.497	74.86 ± 3.398	0.383
		Inferior	64.29 ± 5.752	70.43 ± 6.768	0.535
		Nasal	67.29 ± 5.003	74.43 ± 5.972	0.259
	Outer	Superior	63.14 ± 3.074	62.71 ± 3.496	0.902
		Temporal	67.86 ± 2.375	67.00 ± 1.799	0.710
		Inferior	58.86 ± 2.064	61.29 ± 4.258	0.318
		Nasal	57.29 ± 3.421	65.86 ± 4.554	0.128
RPE	Inner	Superior	15.86 ± 0.769	14.00 ± 0.436	0.073
		Temporal	14.43 ± 0.812	13.86 ± 0.261	1.000
		Inferior	14.57 ± 0.685	13.86 ± 0.340	0.620
		Nasal	14.86 ± 0.884	14.71 ± 0.360	0.710
	Outer	Superior	14.00 ± 0.617	13.00 ± 0.309	0.259
		Temporal	13.29 ± 0.937	11.43 ± 1.251	0.710
		Inferior	12.86 ± 0.705	12.57 ± 0.297	0.710
		Nasal	13.86 ± 0.670	13.29 ± 0.421	0.620

The Mann-Whitney U Test was used to compare non-parametric data. GCL – ganglion cell layer, IPL – inner plexiform layer, INL - inner nuclear layer, ONL - outer nuclear layer, RNFL - retinal nerve fibre layer, RPE – Retinal pigment epithelium, SEM - standard error of the mean. $*P \leq 0.05$; $**P \leq 0.01$; $***P \leq 0.001$.

```
P10589      COT1_HUMAN         1   MAMVVSSWRDPQDDVAGGNPGGPNPAAQAARGGGGGAGEQQ-QQAGSGAPHTPQTPGQPG    59
Q9TTR8      COT1_BOVIN         1   MAMVVSSWRDPQDDVAGGNPGGPNPAAQAARGGGGGAGEQQQQQAGSGAPHTPQTPGQPG    60
A0A5F4DD00  A0A5F4DD00_CANLF   1   MAMVVSSWRDPQD-LAGGNPGGPNPAAAARG-GGGG-GGEQQQQAGSGAPHTPQTPGQPG    57
Q06725      N2F1A_DANRE        1   MAMVVSVWRDPQEDVAGGPPSGPNPAAQPAR------EQ---QQAASAAPHTPQTPSQPG    51
F1NJL8      F1NJL8_CHICK       1   MAMVVSSWRDPQEDVAGGTPSGPNPAAQPAR------EQPPQQQGGSAAPHTPQTPSQPG    54
I2CYU1      I2CYU1_MACMU       1   MAMVVSSWRDPQDDVAGGNPGGPNPAAQAARGGGGGAGEQQ-QQAGSGAPHTPQTPGQPG    59
Q32NY6      Q32NY6_MOUSE       1   MAMVVSSWRDPQDDVAGGNPGGPNPAAQAARGGGGGE----QQQAGSGAPHTPQTPGQPG    56
H2R404      H2R404_PANTR       1   MAMVVSSWRDPQDDVQ-------------------------QQQAGSGAPHTPQTPGQPG    35
F2Z3S9      F2Z3S9_RAT         1   MAMVVSSWRDPQDDVAGGNPGGPNPAAQAARGGGGGE----QQQAGSGAPHTPQTPGQPG    56
A4QNF4      A4QNF4_XENTR       1   MAMVVSSWRDPQEDVAGGNPGGPNPGARE------------QQQAPSAAPHTPQTPSQPG    48
                                   ****** *****: :                          **. *.********.***

P10589      COT1_HUMAN         60  APATPGTAGDKGQGPPGSGQS-QQHIECVVCGDKSSGKHYGQFTCEGCKSFFKRSVRRNL   118
Q9TTR8      COT1_BOVIN         61  APATPGTAGDKGQGPPGSGQS-QQHIECVVCGDKSSGKHYGQFTCEGCKSFFKRSVRRNL   119
A0A5F4DD00  A0A5F4DD00_CANLF  58  APATPGTAGDKGQGPPGSGQS-QQHIECVVCGDKSSGKHYGQFTCEGCKSFFKRSVRRNL   116
Q06725      N2F1A_DANRE        52  PPSTPGTAGDKGS--QNSGQ-SQQHIECVVCGDKSSGKHYGQFTCEGCKSFFKRSVRRNL   108
F1NJL8      F1NJL8_CHICK       55  PPSTPGTAGDKGPGQQGSGQ-SQQHIECVVCGDKSSGKHYGQFTCEGCKSFFKRSVRRNL   113
I2CYU1      I2CYU1_MACMU       60  APATPGTAGDKGQGPPGSGQS-QQHIECVVCGDKSSGKHYGQFTCEGCKSFFKRSVRRNL   118
Q32NY6      Q32NY6_MOUSE       57  APATPGTAGDKGQGPPGSGQS-QQHIECVVCGDKSSGKHYGQFTCEGCKSFFKRSVRRNL   115
H2R404      H2R404_PANTR       36  APATPGTAGDKGQGPPGSGQS-QQHIECVVCGDKSSGKHYGQFTCEGCKSFFKRSVRRNL    94
F2Z3S9      F2Z3S9_RAT         57  APATPGTAGDKGQGPPGSGQS-QQHIECVVCGDKSSGKHYGQFTCEGCKSFFKRSVRRNL   115
A4QNF4      A4QNF4_XENTR       49  PPSTPGAAGDKGQQ--GSGQSQQQHIECVVCGDKSSGKHYGQFTCEGCKSFFKRSVRRNL   106
                                    *:***:*****    .***  **************************************

P10589      COT1_HUMAN         119 TYTCRANRNCPIDQHHRNQCQYCRLKKCLKVGMRREAVQRGRMPPTQPNPGQYALTNGDP   178
Q9TTR8      COT1_BOVIN         120 TYTCRANRNCPIDQHHRNQCQYCRLKKCLKVGMRREAVQRGRMPPTQPNPGQYALTNGDP   179
A0A5F4DD00  A0A5F4DD00_CANLF  117 TYTCRANRNCPIDQHHRNQCQYCRLKKCLKVGMRREAVQRGRMPPTQPNPGQYALTNGDP   176
Q06725      N2F1A_DANRE        109 TYTCRANRNCPIDQHHRNQCQYCRLKKCLKVGMRREAVQRGRMPPTQPNPGQYALTNGDP   168
F1NJL8      F1NJL8_CHICK       114 TYTCRANRNCPIDQHHRNQCQYCRLKKCLKVGMRREAVQRGRMPPTQPNPGQYALTNGDP   173
I2CYU1      I2CYU1_MACMU       119 TYTCRANRNCPIDQHHRNQCQYCRLKKCLKVGMRREAVQRGRMPPTQPNPGQYALTNGDP   178
Q32NY6      Q32NY6_MOUSE       116 TYTCRANRNCPIDQHHRNQCQYCRLKKCLKVGMRREAVQRGRMPPTQPNPGQYALTNGDP   175
H2R404      H2R404_PANTR       95  TYTCRANRNCPIDQHHRNQCQYCRLKKCLKVGMRREAVQRGRMPPTQPNPGQYALTNGDP   154
F2Z3S9      F2Z3S9_RAT         116 TYTCRANRNCPIDQHHRNQCQYCRLKKCLKVGMRREAVQRGRMPPTQPNPGQYALTNGDP   175
A4QNF4      A4QNF4_XENTR       107 TYTCRANRNCPIDQHHRNQCQYCRLKKCLKVGMRRE-VQRGRMPPTQPNPGQYALTNGDP   165
                                   ************************************************************

P10589      COT1_HUMAN         179 LNGHCYLSGYISLLLRAEPYPTSRYGSQCMQPNNIMGIENICELAARLLFSAVEWARNIP   238
Q9TTR8      COT1_BOVIN         180 LNGHCYLSGYISLLLRAEPYPTSRYGSQCMQPNNIMGIENICELAARLLFSAVEWARNIP   239
A0A5F4DD00  A0A5F4DD00_CANLF  177 LNGHCYLSGYISLLLRAEPYPTSRYGSQCMQPNNIMGIENICELAARLLFSAVEWARNIP   236
Q06725      N2F1A_DANRE        169 LNGHCYLSGYISLLLRAEPYPTSRYGSQCMQPNNIMGIENICELAARLLFSAVEWARNIP   228
F1NJL8      F1NJL8_CHICK       174 LNGHCYLSGYISLLLRAEPYPTSRYGSQCMQPNNIMGIENICELAARLLFSAVEWARNIP   233
I2CYU1      I2CYU1_MACMU       179 LNGHCYLSGYISLLLRAEPYPTSRYGSQCMQPNNIMGIENICELAARLLFSAVEWARNIP   238
Q32NY6      Q32NY6_MOUSE       176 LNGHCYLSGYISLLLRAEPYPTSRYGSQCMQPNNIMGIENICELAARLLFSAVEWARNIP   235
H2R404      H2R404_PANTR       155 LNGHCYLSGYISLLLRAEPYPTSRYGSQCMQPNNIMGIENICELAARLLFSAVEWARNIP   214
F2Z3S9      F2Z3S9_RAT         176 LNGHCYLSGYISLLLRAEPYPTSRYGSQCMQPNNIMGIENICELAARLLFSAVEWARNIP   235
A4QNF4      A4QNF4_XENTR       166 LNGHCYLSGYISLLLRAEPYPTSRYGSQCMQPNNIMGIENICELAARLLFSAVEWARNIP   225
                                   ************************************************************

P10589      COT1_HUMAN         239 FFPDLQITDQVSLLRLTWSELFVLNAAQCSMPLHVAPLLAAAGLHASPMSADRVVAFMDH   298
Q9TTR8      COT1_BOVIN         240 FFPDLQITDQVSLLRLTWSELFVLNAAQCSMPLHVAPLLAAAGLHASPMSADRVVAFMDH   299
A0A5F4DD00  A0A5F4DD00_CANLF  237 FFPDLQITDQVSLLRLTWSELFVLNAAQCSMPLHVAPLLAAAGLHASPMSADRVVAFMDH   296
Q06725      N2F1A_DANRE        229 FFPDLQITDQVSLLRLTWSELFVLNAAQCSMPLHVAPLLAAAGLHASPMSADRVVAFMDH   288
F1NJL8      F1NJL8_CHICK       234 FFPDLQITDQVALLRLTWSELFVLNAAQCSMPLHVAPLLAAAGLHASPMSADRVVAFMDH   293
I2CYU1      I2CYU1_MACMU       239 FFPDLQITDQVSLLRLTWSELFVLNAAQCSMPLHVAPLLAAAGLHASPMSADRVVAFMDH   298
Q32NY6      Q32NY6_MOUSE       236 FFPDLQITDQVSLLRLTWSELFVLNAAQCSMPLHVAPLLAAAGLHASPMSADRVVAFMDH   295
H2R404      H2R404_PANTR       215 FFPDLQITDQVSLLRLTWSELFVLNAAQCSMPLHVAPLLAAAGLHASPMSADRVVAFMDH   274
F2Z3S9      F2Z3S9_RAT         236 FFPDLQITDQVSLLRLTWSELFVLNAAQCSMPLHVAPLLAAAGLHASPMSADRVVAFMDH   295
A4QNF4      A4QNF4_XENTR       226 FFPDLQITDQVALLRLTWSELFVLNAAQCSMPLHVAPLLAAAGLHASPMSADRVVAFMDH   285
                                   ***********:************************************************

P10589      COT1_HUMAN         299 IRIFQEQVEKLKALHVDSAEYSCLKAIVLFTSDACGLSDAAHIESLQEKSQCALEEYVRS   358
Q9TTR8      COT1_BOVIN         300 IRIFQEQVEKLKALHVDSAEYSCLKAIVLFTSDACGLSDAAHIESLQEKSQCALEEYVRS   359
A0A5F4DD00  A0A5F4DD00_CANLF  297 IRIFQEQVEKLKALHVDSAEYSCLKAIVLFTSDACGLSDAAHIESLQEKSQCALEEYVRS   356
Q06725      N2F1A_DANRE        289 IRIFQEQVEKLKALHVDSAEYSCIKAIVLFTSDACGLSDAAHIESLQEKSQCALEEYVRS   348
F1NJL8      F1NJL8_CHICK       294 IRIFQEQVEKLKALHVDSAEYSCLKAIVLFTSDACGLSDAAHIESLQEKSQCALEEYVRS   353
I2CYU1      I2CYU1_MACMU       299 IRIFQEQVEKLKALHVDSAEYSCLKAIVLFTSDACGLSDAAHIESLQEKSQCALEEYVRS   358
Q32NY6      Q32NY6_MOUSE       296 IRIFQEQVEKLKALHVDSAEYSCLKAIVLFTSDACGLSDAAHIESLQEKSQCALEEYVRS   355
H2R404      H2R404_PANTR       275 IRIFQEQVEKLKALHVDSAEYSCLKAIVLFTSDACGLSDAAHIESLQEKSQCALEEYVRS   334
F2Z3S9      F2Z3S9_RAT         296 IRIFQEQVEKLKALHVDSAEYSCLKAIVLFTSDACGLSDAAHIESLQEKSQCALEEYVRS   355
A4QNF4      A4QNF4_XENTR       286 IRIFQEQVEKLKALHVDSAEYSCLKAIVLFTSDACGLSDAAHIESLQEKSQCALEEYVRS   345
                                   ***********************:************************************

P10589      COT1_HUMAN         359 QYPNQPSRFGKLLLRLPSLRTVSSSVIEQLFFVRLVGKTPIETLIRDMLLSGSSFNWPYM   418
Q9TTR8      COT1_BOVIN         360 QYPNQPSRFGKLLLRLPSLRTVSSSVIEQLFFVRLVGKTPIETLIRDMLLSGSSFNWPYM   419
A0A5F4DD00  A0A5F4DD00_CANLF  357 QYPNQPSRFGKLLLRLPSLRTVSSSVIEQLFFVRLVGKTPIETLIRDMLLSGSSFNWPYM   416
Q06725      N2F1A_DANRE        349 QYPNQPSRFGKLLLRLPSLRTVSSSVIEQLFFVRLVGKTPIETLIRDMLLSGSSFNWPYM   408
F1NJL8      F1NJL8_CHICK       354 QYPNQPSRFGKLLLRLPSLRTVSSSVIEQLFFVRLVGKTPIETLIRDMLLSGSSFNWPYM   413
I2CYU1      I2CYU1_MACMU       359 QYPNQPSRFGKLLLRLPSLRTVSSSVIEQLFFVRLVGKTPIETLIRDMLLSGSSFNWPYM   418
Q32NY6      Q32NY6_MOUSE       356 QYPNQPSRFGKLLLRLPSLRTVSSSVIEQLFFVRLVGKTPIETLIRDMLLSGSSFNWPYM   415
H2R404      H2R404_PANTR       335 QYPNQPSRFGKLLLRLPSLRTVSSSVIEQLFFVRLVGKTPIETLIRDMLLSGSSFNWPYM   394
F2Z3S9      F2Z3S9_RAT         356 QYPNQPSRFGKLLLRLPSLRTVSSSVIEQLFFVRLVGKTPIETLIRDMLLSGSSFNWPYM   415
A4QNF4      A4QNF4_XENTR       346 QYPNQPSRFGKLLLRLPSLRTVSSSVIEQLFFVRLVGKTPIETLIRDMLLSGSSFNWPYM   405
                                   ************************************************************

P10589      COT1_HUMAN         419 SIQCS                                                          423
Q9TTR8      COT1_BOVIN         420 SIQCS                                                          424
A0A5F4DD00  A0A5F4DD00_CANLF  417 SIQCS                                                          421
Q06725      N2F1A_DANRE        409 SIQ--                                                          411
F1NJL8      F1NJL8_CHICK       414 SIQCS                                                          418
I2CYU1      I2CYU1_MACMU       419 SIQCS                                                          423
Q32NY6      Q32NY6_MOUSE       416 SIQCS                                                          420
H2R404      H2R404_PANTR       395 SIQCS                                                          399
F2Z3S9      F2Z3S9_RAT         416 SIQCS                                                          420
A4QNF4      A4QNF4_XENTR       406 PIQCS                                                          410
                                    **
```

Supplementary Figure 1. Multiple alignment of human NR2F1 orthologues. Multiple alignment shows strict conservation across different species. Missense variants and small deletions have been indicated in red shading. Loss of the c-terminal 24 amino acid residues has been indicated in blue shading.

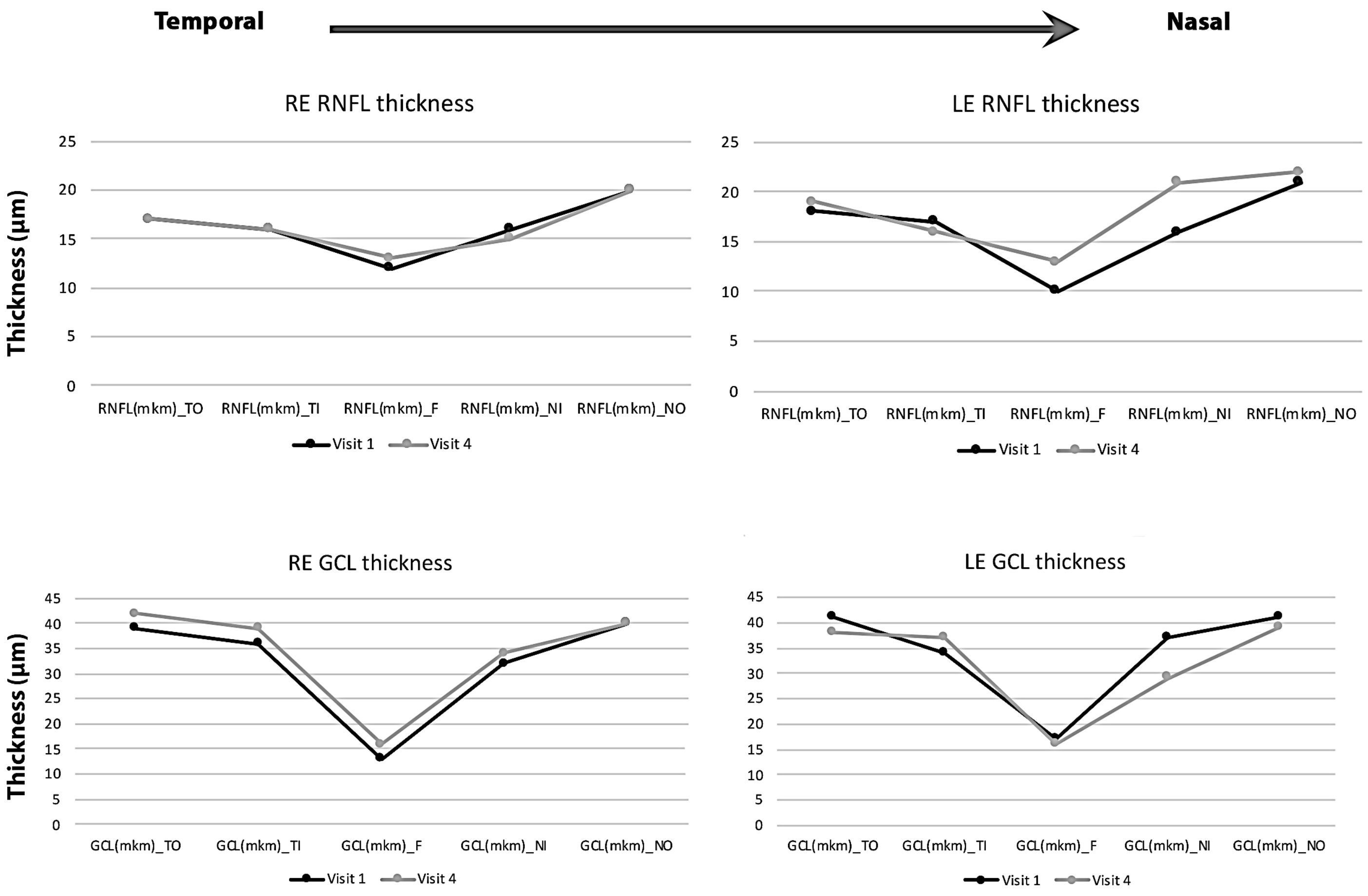

Supplementary Figure 2. Change in RNFL and GCL thickness during long-term follow-up for subject NR2F1_14 (UK). No marked change in RNFL and GCL thickness was observed between Visit 1 and Visit 4, which were 5 years months apart. F – foveal; GCL – ganglion cell layer; NI – nasal inner; NO – nasal outer; RNFL – retinal nerve fibre layer; TI - temporal inner; TO – temporal outer.

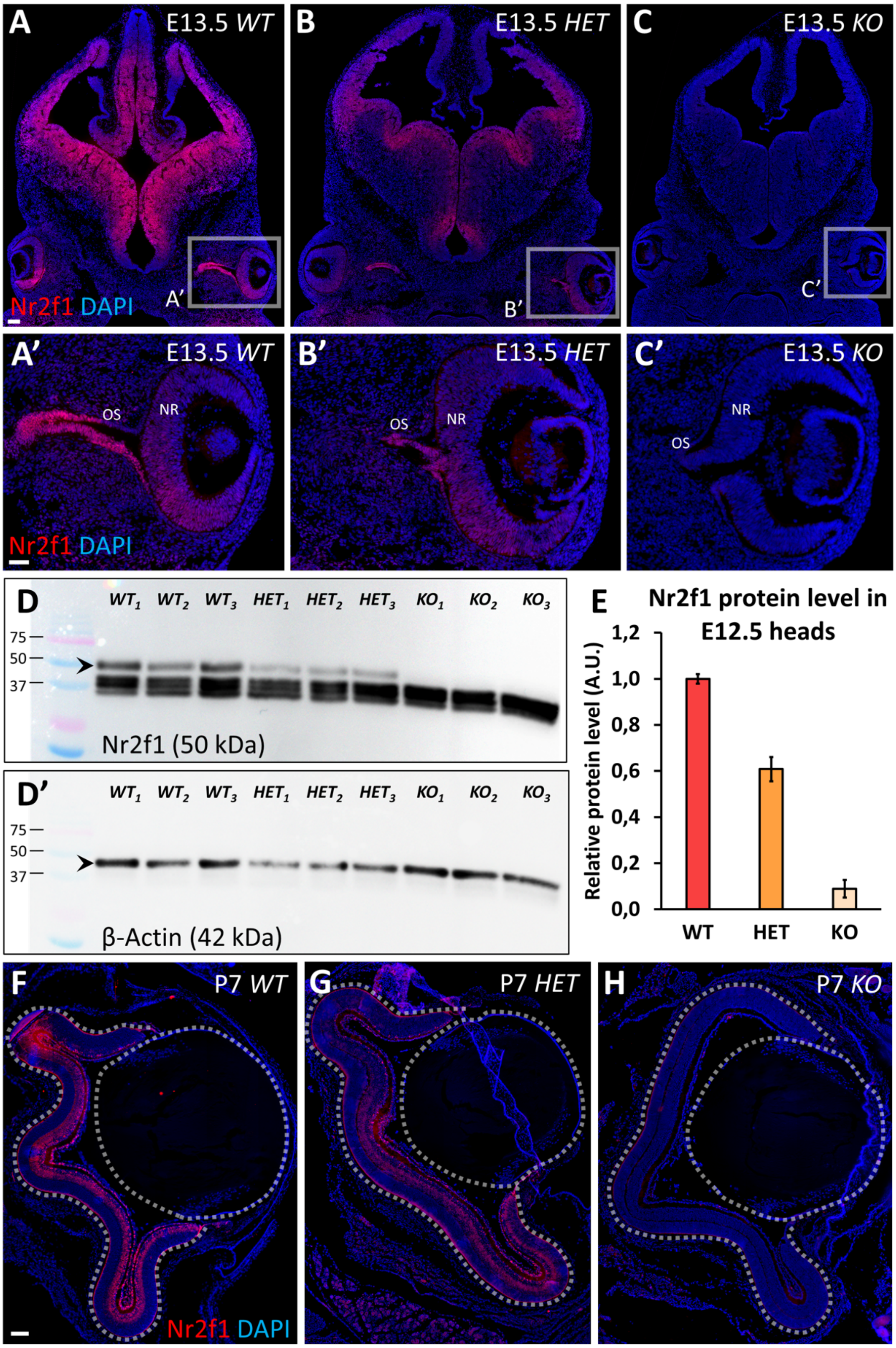

Supplementary Figure 3. Decreased or abolished Nr2f1 protein levels in an *Nr2f1* mutant mouse model. (A-B) Nr2f1 (red) immunofluorescence (IF) of head cross-sections from wild-type (*WT*; A-A'), heterozygous (*HET*; B-B') and knock-out mutant (*KO*; C-C')

mice at embryonic day (E) 13.5. Genetic loss of one (*HET*) or two *Nr2f1* alleles (*KO*) resulted in decreased (B,B') or absent (C,C') Nr2f1 protein compared with the physiological situation (A-A'). Magnifications show Nr2f1 in the eye region (A'-C'), comprising the neural retina (NR) and the optic stalk (OS). (D-E) Western blot (WB) quantification of Nr2f1 protein at embryonic day (E) 12.5 from embryo head extracts. Nr2f1 detection is shown in panel D (predicted band size: 46 kDa; observed band size: 50 kDa), while the housekeeping protein β-Actin, which was used for normalization, is shown in panel D'. Mutant E12.5 embryo heads comprising eyes, optic nerves and brains, contained decreased (*HET*) or abolished (*KO*) amount of Nr2f1 protein compared with *WT* ones. (E) Signal quantification after revelation of WB membrane. *HET* embryos display half quantity of Nr2f1 protein compared with *WT*, suggesting that loss of one allele results in protein haploinsufficiency. The complete absence of signal in *KO* lines indicates complete genetic removal of Nrf21 expression. (F-H) Nr2f1 (red) IF at post-natal (P) day 7 of retina from wild-type (*WT*; F), heterozygous (*HET*; G) and *Nr2f1* knock-out mutant (*KO*; H) mice. Nr2f1 expression in P7 retinas, which was indirectly visualized by immunostaining, showed a proportional change depending on the animal's genotype. The nuclei (blue) were stained with DAPI. Scale bars: 50 μm, except A-C (100 μm). NR – neural retina; OS – optic stalk.

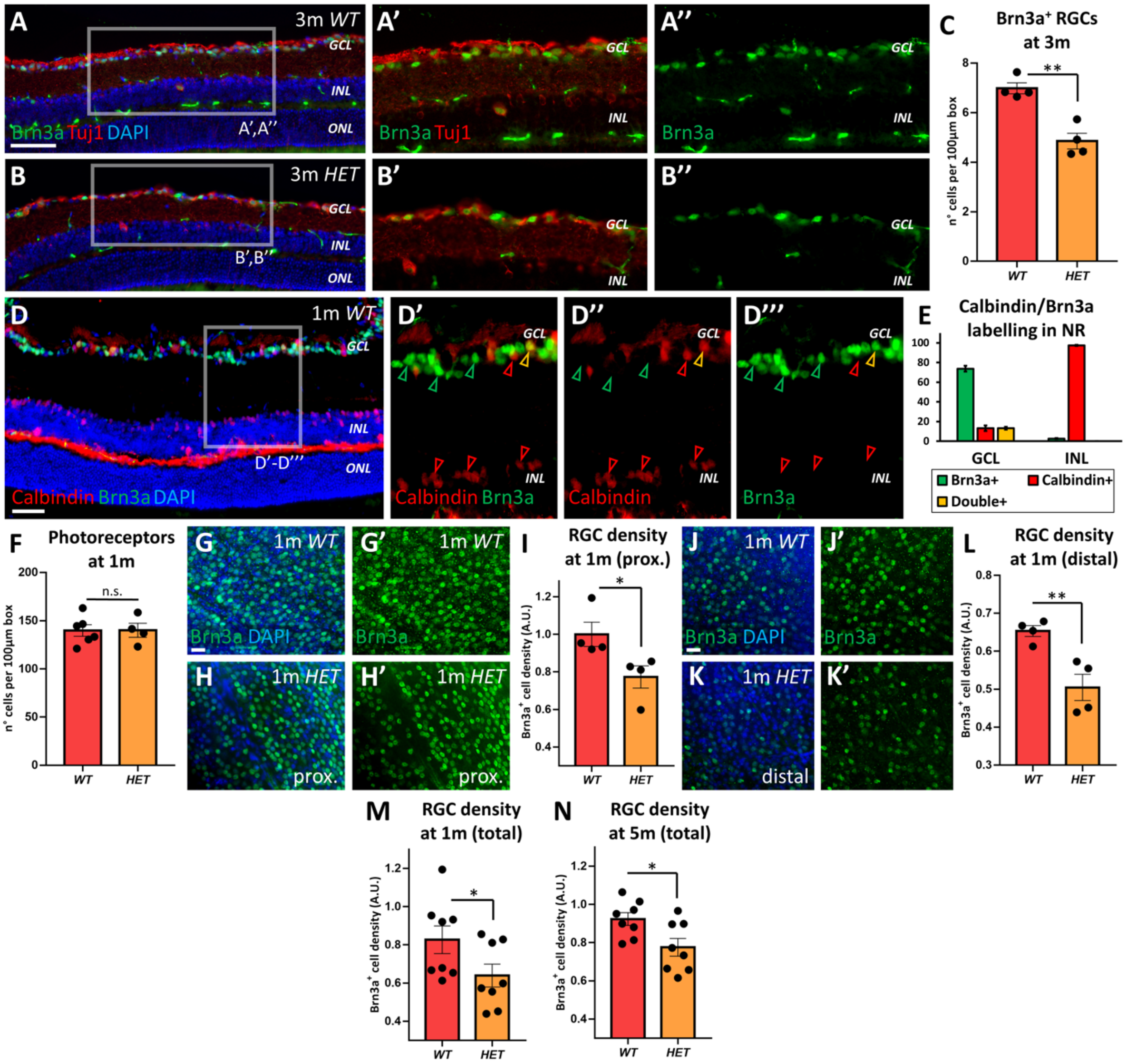

Supplementary Figure 4. Altered retinal development in Nr2f1-deficient mice. (A-C) Brn3a (green; RGCs) and Tuj1 (red) immunofluorescence (IF) of retinal sections from three-month-old (3m) *WT* (A-A'') and mutant *HET* (B-B'') mice. Partial *Nr2f1* loss resulted in decreased number of Brn3a^{+} RGCs. (C) Quantification of RGC density in the GCL (*WT/HET*: **=0.0015). (D-E) Brn3a (green; RGCs) and Calbindin (red) double IF on one-month-old (1m) *WT* retinal sections. The arrowheads (D'-D''') point to Calbindin^{+} (red arrowheads), Brn3a^{+} (green arrowheads) and double Calbindin^{+}Brn3a^{+} cells (yellow arrowheads). (E) Co-localisation and quantification of Calbindin and Brn3a in the INL and GCL. Double-labelled cells were found in the GCL (13.16±1.34%), but not in the INL. (F) ONL density photoreceptors (PR) quantified in retina from one-month-old *WT* and mutant *HET* mice (*WT/HET*: n.s.=0.98). (G-M) Whole mount IF with Brn3a (green; RGCs) in retinas from one-month-old (1m) *WT* (G,G',J,J') and mutant *HET* (H,H',K,K') mice. The density of

Brn3a$^+$ RGCs in proximal (G-H') and distal (J-K') retinal regions or in the whole retinal surface (total) is shown in panels I, L and M, respectively (*WT* total /*HET* total: P=0.03272; *WT* proximal /*HET* proximal: P=0.0415; *WT* distal / *HET* distal: P=0.0073). (N) Graph shows the density of Brn3a$^+$ RGCs in five-month-old *WT* and *HET* retinas (*WT* total /*HET* total: P=0.0214); proximal and distal retinal regions from five-month-old animals are shown in Figures 5M-S. In graphs C, E, and F, the number of positive cells was quantified in 60µm-width boxes, randomly placed across the NR. In graphs I, L, M and N, the number of Brn3a$^+$ cells was counted within squares of 100µm-width regions, randomly placed in the central (proximal) or peripheral (distal) regions of flattened whole-mount retinal explants. Brn3a$^+$ cell number was normalized on the average RGC density in *WT* retinas (M-N). The nuclei (blue) were stained with DAPI. The data have been represented as mean ± standard error of the mean (SEM); n = 4 retinas from 2 animals per genotype, except F (n = 6 retinas from n = 3 animals per genotype). The Student t-test was used for statistical analysis (C, F, I, L, M and N) (*P<0.05; **P<0.01). Scale bars: 50 µm. A.U. – arbitrary unit; GCL – ganglion cell layer; INL – inner nuclear layer; NR – neural retina; ONL – outer nuclear layer; RGC – retinal ganglion cell.

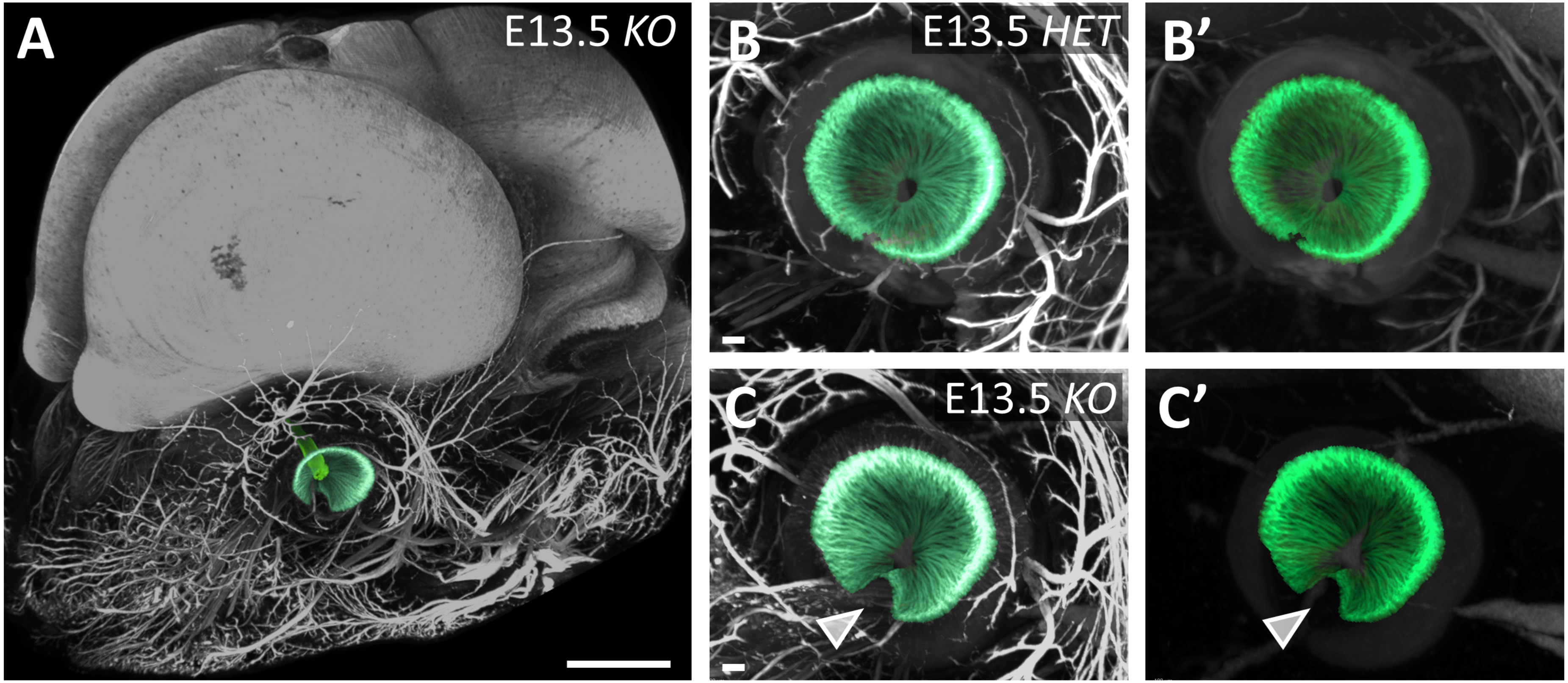

Supplementary Figure 5. Three-dimensional (3D) reconstruction of the developing retina and optic nerve in *Nr2f1* mutants. (A) 3D imaging of a whole E13.5 *KO* (*null*) embryo head after Tuj1 immunofluorescence and tissue clarification. A false green colour has been used to highlight the retina (dark green) and the ON (light green). (B-C’) 3D visualization of $Tuj1^{+}$ axonal fibres in the retina (green) in *HET* (B-B’) and *KO* (C-C’) eyes of E13.5 embryos. The white arrowheads in (C-C’) point to an invagination of the ventral retina in the *KO* eye. Scale bars: 300 µm in A, 30 µm in B,C.

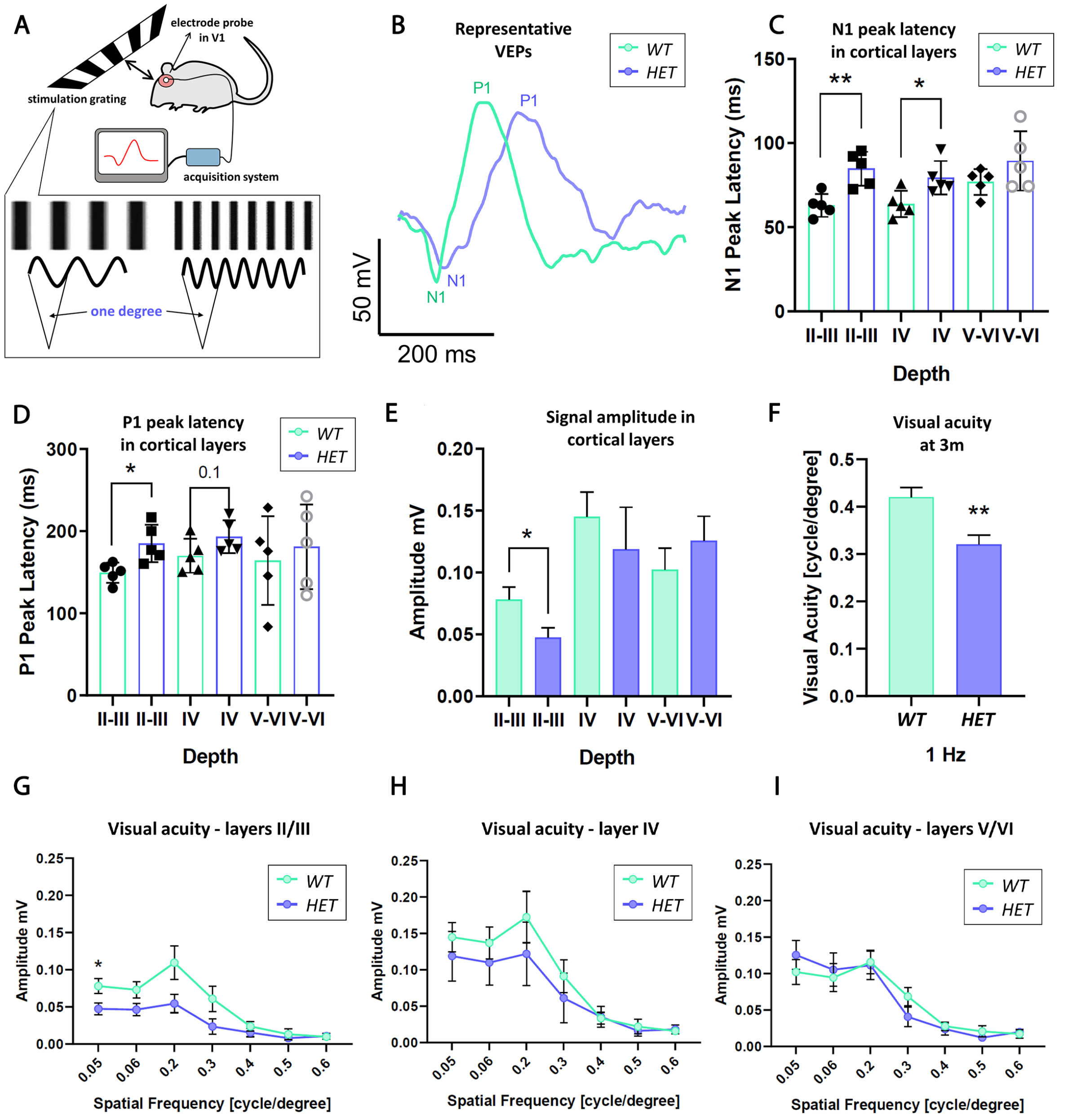

Supplementary Figure 6. Altered visual acuity and VEP signal amplitude in the V1 cortical layers in an *Nr2f1* mutant mouse model. (A) Schematics showing the experimental approach for electrophysiological analysis of visual acuity in mice. Silicon probes (16 channels) spanning the whole thickness of the primary visual cortex (V1) were used to record visual evoked potentials (VEPs) following stimulation with gratings of increasing spatial frequencies, ranging from 0.05 to 0.6 cycles/degree (contrast 30% and temporal frequency of alternation 1 Hz). Spatial frequency is a measure of the number of cycles subtended at the eye per degree (lower schematics). (B) Representative VEPs recorded in the superficial cortical? layers in *WT versus HET* mice. (C-D) VEP latency of the major negative (N1; C) and positive (P1; D) components in three-month-old (3m) *WT* and *HET* animals and in different cortical

layers, as indicated. Increased signal latency was recorded in superficial cortical layers of *HET* animals, suggesting lower conduction velocity along the visual pathway. (E) VEP amplitude (mV), at 0.05 cycles/degree and 30% contrast, of *WT versus HET* 3m animals in different cortical layers, as indicated. Significantly decreased amplitude was found in superficial layers of visual cortex of mutant animals. (F) Electrophysiological assessment of visual acuity in 3m *WT* and mutant *HET* mice. Visual acuity is significantly reduced upon loss of one *Nr2f1* allele. Separately recorded amplitudes in *WT* and *HET* animals in distinct layers and in response to gratings of increasing spatial frequencies are shown in G-I. (G-I) VEP amplitude of three-month-old *WT* versus *HET* animals in different cortical layers, as indicated. Significantly decreased amplitude was found in the superficial layers of the visual cortex of mutant animals. The graphs show recorded amplitudes in layers II/III (G), layer IV (H), and layers V/VI (I) in *WT* and *HET* animals in response to gratings of increasing spatial frequencies as indicated. The data has been represented as mean ± standard error of the mean (SEM). The 2-way ANOVA was used for statistical analysis (*$P \leq 0.05$; **$P \leq 0.01$); n = 5 animals per genotype.
